# Regular collagen peptide administration exerts anti-obesity effects in high-caloric diet-fed rodents—a systematic review with meta-analysis of animal trials

**DOI:** 10.1038/s41366-025-01905-3

**Published:** 2025-10-30

**Authors:** Kevin Bischof, Anna Maria Moitzi, Daniel König

**Affiliations:** 1 Centre for Sports Science and University Sports, Department of Sports Science, Section for Nutrition, Exercise and Health, University of Vienna, Vienna, Austria; 2 Vienna Doctoral School of Pharmaceutical, Nutritional and Sport Sciences, University of Vienna, Vienna, Austria; 3 Faculty of Life Sciences, Department for Nutrition, Section for Nutrition, Exercise and Health, University of Vienna, Vienna, Austria

**Keywords:** Preclinical research, Obesity

## Abstract

**Background:**

Cost-effective and easy-to-implement nutritional approaches to overcoming obesity and accompanying diseases remain of considerable therapeutic interest. Collagen peptides (CP) have previously demonstrated potential to alleviate obesity and related symptoms during high-fat/ high-caloric diets. Therefore, a systematic review and meta-analysis have been conducted to pool the evidence from the last decades.

**Methods:**

A systematic literature search was conducted in accordance with the PRISMA statement in PubMed, Scopus, CINAHL, MEDLINE and ScienceDirect in March 2024. Risk of bias was assessed using the SYRCLE tool for animal studies. A random-effects model with standardized mean differences (SMD) calculated overall effect sizes. Preclinical controlled trials as well as randomized controlled trials were eligible if rodents received a high-fat or high-caloric diet, were genetically not modified and administered CPs compared to a placebo for at least 3 weeks.

**Results:**

Twenty-one and nineteen (*n* = 339) studies were included in the systematic review and meta-analysis, respectively. A several-week CP supplementation led to significant decreases in body mass (*p* < 0.01; SMD = −1.87), food intake (*p* = 0.01; SMD = −1.43), glucose metabolism (*p* < 0.02; SMD = −2.21), adipose tissue & organ content (*p* < 0.01; SMD = −1.26), LDL (*p* < 0.01; SMD = −2.02), triacylglycerol (*p* < 0.01; SMD = −2.16) and leptin (*p* = 0.01; SMD = −1.33). Significant increases were observed in HDL (*p* = 0.04; SMD = 1.14) and adiponectin (*p* < 0.01; SMD = 1.04). A moderate to high risk of bias, combined with a very low certainty of evidence for each parameter, was apparent.

**Conclusion:**

CP administration in rodents with obesity for at least 3 weeks demonstrated significant anti-obesity effects by positively influencing body mass, food intake, glucose metabolism, lipid markers, adipose tissue and adipokines. However, CP’s anti-obesity effects in humans remain largely unexplored, necessitating further research.

## Introduction

According to the World Obesity Federation, ~40% of the global population currently faces overweight or obesity, predicting to reach a yet unprecedented milestone of over 50% in 2035 [1]. If the trend of increasing obesity rates persists, it is anticipated that there will be significant economic costs amounting to approximately 3.3% of the global gross domestic product by 2060 [2]. Obesity, expressed as an elevated body mass index above 29.9, increases the risk of non-communicable diseases such as diabetes mellitus type II (DMT2), cardiovascular diseases and musculoskeletal disorders, leading to a significant decline in both quality of life and life expectancy. As the current rationale for obesity constitutes an excessive caloric intake leading to an overall energy imbalance/surplus, the pathophysiology appears to be of a multifactorial nature [3]. Mechanisms responsible for overfeeding, ectopic lipid accumulation, brain-associated reward regulation, as well as social and physical factors are presently of research interest. Moreover, little is known about genetic and epigenetic causes and the influence on and of the gut microbiome, which may play a pivotal role in obesity [4]. Approaches to adjust individual lifestyles by means of nutritional modifications have been demonstrated to effectively aid in obesity management [5]. Both the amount and source of proteins and even their inherent peptides could be viewed as a prospective therapeutic strategy for influencing body composition, markers of metabolic syndrome and appetite regulation in patients with obesity [6].

Of late, collagen peptides (CP), being unique in their amino acid composition (mainly comprised of glycine, proline, hydroxyproline) and usually received from connective tissues of marine or mammalian sources, have elicited interest in obesity management research. The majority of studies conducted as animal trials have addressed the potential alleviation of metabolic dysregulation associated with high-caloric diet (HCD)-fed rodents in addition with CP administration [7]. Absolute body weight over time compared to a HCD control group has been reported to be lower in Wistar rats supplemented with fish CPs for 2 months, while food intake remained the same [8]. A study applying CPs for 9 weeks, alongside additional fish-derived extracts, demonstrated improved glucose sensitivity during a glucose tolerance test, slight changes in relative abdominal fat deposition, and reduced leptin mRNA expression levels compared to a control high-fat diet (HFD). These findings suggest that CPs may contribute to improved hunger-satiety regulation, help combat leptin resistance in organisms facing obesity, and enhance insulin sensitivity [9]. Lipid serum markers such as total and low-density lipoprotein (LDL) cholesterol and triacylglycerol, which are commonly elevated (= dyslipidemia) in metabolic syndrome and patients with obesity [10], also being predictors of cardiovascular diseases [11], have been reported to be lower in fish CP + HFD-fed mice after 8 weeks [12, 13].

Some of the yet investigated molecular mechanisms involved in obesity include transcription factors that strictly control energy expenditure and storage. Peroxisome proliferator-activated receptor gamma (PPARγ), recognized as one master regulator of adipogenesis, and CCAAT-enhancer-binding proteins (C/EBPs) regulate differentiation of preadipocytes and mesenchymal stem cells to mature adipocytes, based on physiological lipid concentrations. Another transcription factor, Sterol regulatory element-binding proteins (SREBPs), enhances transcription of genes responsible for triglyceride and fatty acid synthesis, which is induced by insulin and therefore also hinged on glucose metabolism [14]. Gene expression levels of PPARγ and C/EBPs have previously been shown to decrease in mouse 3T3-L1 pre-adipocytes following CP addition for 8 days [12]. Likewise, hepatic protein expression of the isoforms SREBP-1 and SREBP-2 (responsible for cholesterol synthesis) was both significantly reduced in C57BL6/J male mice after an 8-week CP administration, conveying a trend towards decreased levels with increased dosages [15].

Obesity induced systemic inflammation has been reported to promote insulin resistance and DMT2-associated complications such as retinopathy and cardiovascular disease [16]. Thus, therapeutic approaches to reduce obesity related inflammatory states are crucial to halt the development of concomitant diseases. One of numerous anti-inflammatory proteins illustrates the insulin-sensitizing adiponectin that hinders maturation of pre-adipocytes to adipocytes [17]. Together with common pro-inflammatory proteins like tumor necrosis factor alpha (TNFα) and C-reactive protein (CRP), the addition of high-dose marine CPs for 1 month revealed positive effects represented by declines in TNFα & CRP and increases in adiponectin expression in type II diabetic rats [18]. Strong anti-inflammatory activity was also observed in apolipoprotein E-deficient mice by two specific CPs derived from salmo sala collagen (~1.2 kDa) by means of a nano-liquid chromatography–tandem mass spectrometry, depicting probable atherosclerosis inhibition effects [19]. Since inflammation often appears to be the cause of augmented oxidative stress due to an upregulation of genes involved in inflammatory pathways [20], CPs have also been reported to deliver antioxidant activity. In obesity-induced db/db mice, an 8-week skate CP supplementation intervention led to reduced concentrations of oxidative stress-related markers and increased the concentration of antioxidants, thereby reducing obesity-induced oxidative stress [21]. Similar results were also obtained in a linoleic acid model system, demonstrating autooxidation inhibition and effective scavenging of radicals [22]. CPs from milkfish even protected against UV radiation and H_2_O_2_-induced DNA single-strand breaks in an in vitro model of human keratinocytes [23].

Although of a correlational nature, recent evidence suggests that the gut microbiome majorly influences energy homeostasis and body mass regulation. HFDs and high-sucrose diets have been shown to induce negative alterations in specific gut microbiota, such as *Firmicutes* to *Bacteroidetes*, where a higher ratio (F/B) of these is usually found in subjects with obesity [24]. In a study administering two CPs from different marine sources over 2 weeks to Sprague Dawley rats, the F/B ratio remained either equal or got lower compared to a normal non-HFD group [25]. In contrast, an increase in the F/B ratio has been reported in CP-receiving mice following a 12-week HFD [26]. Based on these ambiguous findings, more evidence is needed to clarify the interplay between specific diets and the host’s microbial composition.

Therefore and according to the previously mentioned issues, this systematic review and meta-analysis investigates the potential anti-obesity impact of CP supplementation on body mass, food intake, lipid and glucose metabolism, antioxidant capacity, anti-inflammatory status and the gut microbiome of rodents.

## Methods

The current systematic review and meta-analysis were registered in advance on the Open Science Framework (Registration: 10.17605/OSF.IO/38G7F) and developed following the Preferred Reporting Items for Systematic reviews and Meta-Analyses (PRISMA).

### Eligibility criteria

Experimental preclinical trials investigating mice and rats with exercise interventions lasting more (or equal) than 3 weeks were considered for inclusion. Studies conducting trials with genetically modified/ knock-out rodents (e.g., ApoE-/-), in vitro and ex vivo models were excluded. Since anti-obesity-related effects were of interest in the current review, both the treatment and the control group had to follow a high-calorie or high-fat diet. Regardless of its origin and manufacturer, the treatment group additionally received CP regularly. Alcohol, streptozotocin (to induce a T2DM model), additional vitamins, amino acids or other potentially confounding bioactive peptides were prohibited. If studies were published in other languages than English, ChatGPT was used for translation to check for suitability. Moreover, animal- preclinical controlled trials as well as randomized controlled trials were eligible for inclusion, whereas case control and observational studies were excluded.

### Search strategy

Comprehensive literature search was performed in April 2024 using PubMed, EBSCOhost (CINAHL, MEDLINE), Scopus and ScienceDirect. The search strings were as follows: collagen peptide* AND (supplementation* OR administration* OR effect* OR intake OR consumption) AND (“anti-obesity” OR “anti obesity” OR obesity OR adiposity OR adipose OR obese OR fat) AND (animal* OR rodent* OR mice OR mouse OR rodentia OR rat*) AND (“high-fat diet” OR “high fat diet” OR “high caloric diet” OR “high calori diet” OR “high-caloric diet” OR “high-calori diet”). An additional filter in Scopus was used (“Article title, Abstract, Keywords” instead of “All fields”). Due to a restriction to a maximum of eight boolean operators, an adapted search string was applied for ScienceDirect: collagen peptide AND (“anti-obesity” OR “anti obesity”) AND (rodent OR mice OR rat OR animal) AND (high fat diet OR high calori). We conducted a screening of references from the included studies and also utilized Google Scholar for both forward and backward searches. If studies met the eligibility criteria during this process, they were “handpicked” and eligible for inclusion. While gray literature was not actively pursued, it was taken into consideration if it met the eligibility criteria. Following the initial search string query across all databases, literature screening was independently conducted by two reviewers (AMM, KB) using the free online Rayyan tool (https://www.rayyan.ai/). Any discrepancies in the inclusion or exclusion of articles during title, abstract, and full-text screening were resolved through consensus between the two reviewers. If consensus could not be reached, a third reviewer (DK) made the final decision. Data from included studies were primarily collected by KB directly from the articles or through requests to the authors.

### Data collection

The number of animals per group, study design, strain, age, dose, duration of supplementation phase, control diet, origin of CP and outcome parameters were extracted from the included studies and are presented in Table S1. Since several studies included more than a single treatment group, all dosages were listed, but only the highest dose of each trial was considered for meta-analysis. If the required data were not available within the articles, corresponding authors were contacted via email and/or Researchgate to request the information. If authors did not responded, mean and standard deviation (SD) values were extracted from graphs using WebPlotDigitizer (https://automeris.io/WebPlotDigitizer/). If only standard error (SE) instead of SD was provided, SD was calculated using the formula $\mathrm{SD}=\mathrm{SE}* \sqrt{N}$ [27]. For the meta-analysis, individual parameters for each study were selected for the following items. *Body mass*, *food/energy intake*, *adipocyte size* (adipocyte diameter and cross-sectional area), *adipose tissue and organ content* (dorsal fat, subcutaneous (white) adipose tissue (WAT), epididymal WAT, visceral adipose tissue, liver and liver index (weight/body weight in %)), *glucose metabolism* (glucose, insulin, HbA1c), *serum lipids* (total cholesterol, LDL, HDL, triacylglycerol), *oxidative stress* (TBARS, SOD, GPx, CAT, GSH, MDA, ROS), *inflammation* (TNFα, IL1β, IL4, IL6, IL10, IL12, NFκB), *adipokines* (adiponectin, leptin) and *lipid metabolism related transcription factors* (PPARα, PPARγ, SREBP1 & 2, C/EBPα). Regarding *gut microbiome* (firmicutes/ bacteroidetes ratio), one out of three relevant trials depicted mean ± SD, for the remaining two, only the average without SD has been obtained. Due to the low number of data, *the gut microbiome* did not enter the meta-analysis, but was included in the systematic review.

### Risk of bias and certainty of evidence assessment

To assess the risk of bias in preclinical trials including animals, the SYstematic Review Centre for Laboratory animal Experimentation (SYRCLE) tool was utilized as an adapted version of the Cochrane RoB tool, which is usually applied in systematic reviews of human RCTs [28]. The SYRCLE tool examines biases related to selection, performance, detection, attrition, reporting and other sources of bias. In total, 10 items are represented and each can be rated by “yes”, “unclear” or “no”, where a rating high in “yes” scores is indicative of low risk of overall bias. As recommended by the authors [28], a total score for each study was not calculated. Rather, an individual approach was taken, where overall differences and similarities among studies were inspected and interpreted. The methodological evaluation of the included studies was conducted by AMM and KB, with any disagreements resolved by a third reviewer, DK. Moreover, publication bias risk in parameters containing more than ten studies/data sets was assessed using contour-enhanced funnel plots and Egger’s test. The certainty of evidence was evaluated using the Grading of Recommendations Assessment, Development, and Evaluation (GRADE) [29]. Given that all the studies included were randomized controlled trials (RCTs) or at least controlled trials in a parallel group design manner, the grading initially began at a level of “high” certainty. However, outcomes had the potential to be downgraded to “very low” certainty due to various factors such as risk of bias, inconsistency, indirectness, imprecision, and publication bias. In addition, an upgrade was not possible within these study types.

### Meta-analysis

Meta-analyses have been conducted in R (version 4.2.3) using the “metafor” (v. 4.8-0) and “meta” (v. 8.0-2) packages. The analyses utilized the standardized mean difference (SMD), including change scores (post–pre values, resulting in Δmean and ΔSD) and post values gauged in different units/scales since several authors did not respond to data requests. A random-effects model was applied across all variables, with effect sizes expressed as SMD (Hedges’ g) and a 95% confidence interval, illustrated by means of forest plots. Multilevel meta-analysis has been carried out (with “metafor”) for “glucose metabolism”, “inflammation”, “oxidative capacity”, “adipose tissue & organ content” and “lipid metabolism related transcription factors” since studies with multiple variables were included, and therefore, independency between estimators was not given anymore. For all other parameters, a conventional meta-analysis has been applied (with “meta”). The restricted maximum-likelihood was chosen as the model estimator. Heterogeneity was assessed through *I*² (where 25%, 50%, and 75% are considered low, moderate, and high variance, respectively) [30] and Cochran’s Q-statistic (with *p* < 0.05 indicating studies do not share a common effect size [31]). If biomarkers in a specific analysis exhibit opposite beneficial effects (e.g., low ROS and high SOD indicating enhanced antioxidant capacity), signs have been reversed (+ to – and vice versa). The individual changes can be found in the descriptions of the relevant forest plots. Publication bias is usually checked if 10+ studies are involved in a meta-analysis, but we checked all parameters by visually inspecting contour-enhanced funnel plots (Supplementary file 1) and by calculating the *p*-values of Egger’s regression test (*p* < 0.05 implying potential publication bias). Subgroup analysis was performed to explore origins of heterogeneity for body mass due to having included the most studies (*n* = 18) of all parameters. Factors examined were species (rats/mice), intervention duration (≤ 6 weeks, > 6 weeks), age (< 8 weeks, ≥ 8 weeks; since rats are considered adult after approximately 8 weeks [32]) and CP source (marine/mammals).

## Results

Out of 374 articles obtained from the initial search, 18 were assessed for eligibility (Fig. S1). Three of them were excluded due to not providing the full text, inappropriate diet design [8] (control and treatment group were additionally substituted with alcohol) and genetically modified (apolipoprotein E-deficient (ApoE-/-)) mice [19]. Identification of studies via other methods (websites (Google Scholar) and citation searching, e.g., citation mining, forward and backward citation search) led to eleven articles from which five were excluded because of genetic modification (db/db rats), streptozotocin-induced type 2 diabetes mellitus, no full-text provision, CP supplementation mixed with cod(-fish) powder and absence of high-caloric/-fat diet. In total, the systematic review comprises 21 articles (*n* = ~354, one article did not state “n” [33], none of them considered as gray literature) and 19 were eligible for meta-analysis (*n* = 338). Ten trials were conducted in a parallel group design whereas the remaining eleven applied randomization. Rodent strains involved Sprague Dawley rats (*n* = 5), Wistar rats (*n* = 3), C57BL/6 J mice (*n* = 10), ddY mice (*n* = 1), ICR mice (*n* = 1) and BALB/c mice (*n* = 1). Rodent age ranged from 5 to 13 (average of 7.7) weeks after acclimatization at the start of the experimental intervention. Supplementation phase lasted 3 to 20 weeks with an average of 2 months. Nineteen studies provided high-fat or HDCs. One study used a normal control diet [34] and one trial did not state the type of diet [35], but due to appropriate study designs, we decided to include them. The majority of experiments produced and/or administered marine-derived CPs, the minority being extracted from porcine, sheep, chicken, bovine and yak sources.

### Body mass and subgroup analysis

**Fig. 1:**
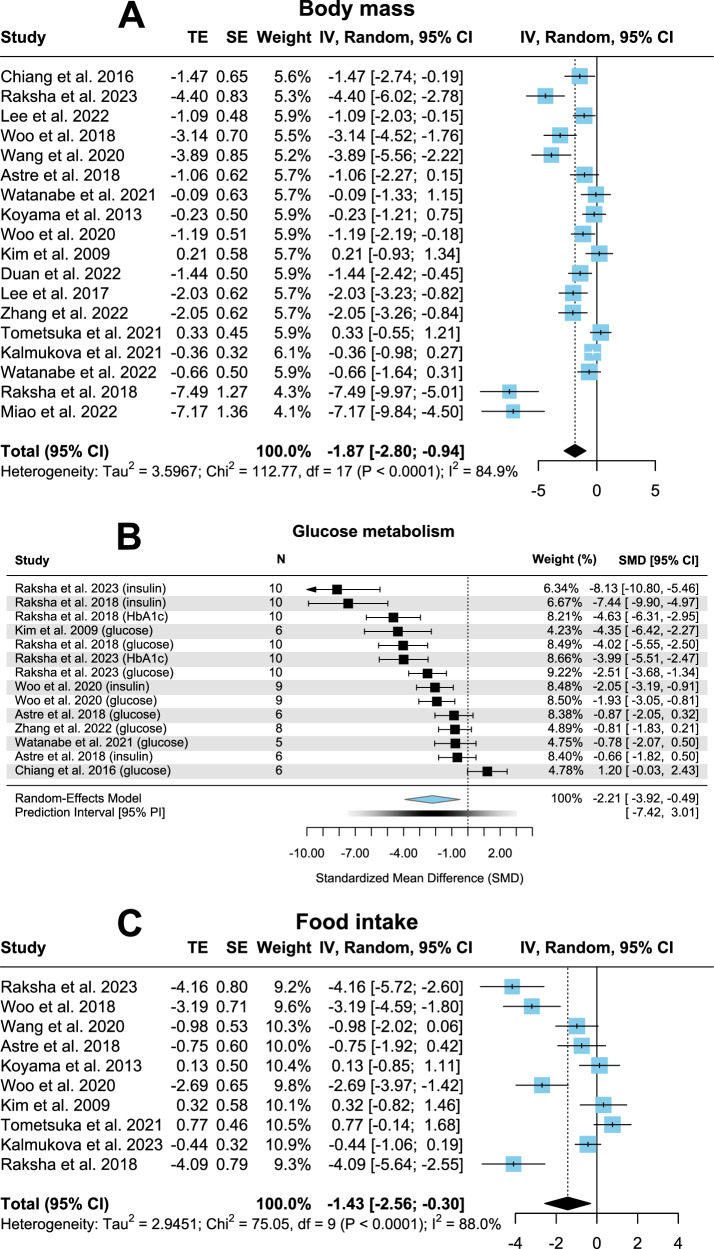
Forest plots illustrating body mass, glucose metabolism and food intake with pooled estimates, study weights and confidence intervals. **A** Body mass. **B** Glucose metabolism. **C** Food intake.

Eighteen studies (*n* = 318) investigated CPs influence on rodent’s body mass. As seen in Fig. 1A and Table 1, body mass has been reduced significantly in the CP group (*p* < 0.01; SMD = −1.87; CI = −2.8, −0.94). High heterogeneity was observed (*I*² = 84.9%) as well as potential publication bias (*p* < 0.01, Fig. S2). Subgroup analysis (Table 2) showed no significant changes when correcting for species, intervention duration and age. CP source influenced heterogeneity when studies administering fish-derived CP (and studies which did not provide data on the source) were excluded, reaching an *I*² of 53.9% (moderate). Simultaneously, SMD changed to −0.6 and *p* = 0.07 with a CI of −1.25, 0.06. Therefore, supplement “type” as a particular subgroup reached significance of *p* = 0.02, implying supplement “type” to be a potential source of heterogeneity.

**Table 1:** Estimated standardized mean differences (SMD), heterogeneity statistics (*τ*², *I*², *Q*), publication bias assessment (by means of Egger’s regression test) and certainty of evidence ratings.

Parameter	No. of studies	No. of subjects	Estimate	*p*	*I*² (%)	*Q*	*p* (Q)	Egger’s regression test (*p*)	Certainty of evidence (GRADE)
**Body mass**	18	318	−1.87	**<0.01**	84.9	112.8	<0.01	<0.01	**⨁◯◯◯** **very low**
**Food intake**	10	192	−1.43	**0.01**	88	75	<0.01	0.02	**⨁◯◯◯** **very low**
**Glucose metabolism**	8	120	−2.21	**0.02**	91.6	110.7	<0.01	<0.01	**⨁◯◯◯** **very low**
**Adipose tissue & organ content**	10	176	−1.26	**<0.01**	75.2	94.5	<0.01	<0.01	**⨁◯◯◯** **very low**
Adipocyte size	5	112	−2.21	0.053	91.4	46.7	<0.01	0.3	⨁◯◯◯ very low
Total cholesterol	12	188	−1.49	0.08	90.8	119.5	<0.01	0.39	⨁◯◯◯ very low
**LDL**	7	118	−2.02	**<0.01**	80.2	30.4	<0.01	0.01	**⨁◯◯◯** **very low**
**HDL**	6	98	1.14	**0.04**	81.8	27.4	<0.01	0.04	**⨁◯◯◯** **very low**
**Triacyglycerol**	13	204	−2.16	**<0.01**	87.9	99.5	<0.01	<0.01	**⨁◯◯◯** **very low**
**Adiponectin**	4	64	1.04	**<0.01**	22.3	3.9	0.28	0.6	**⨁◯◯◯** **very low**
**Leptin**	4	64	−1.33	**0.01**	65.6	8.7	0.03	0.21	**⨁◯◯◯** **very low**
Oxidative capacity	7	120	1.83	0.16	98.5	362.6	<0.01	0.09	⨁◯◯◯ very low
Inflammation	5	80	−3.82	0.22	99.1	157.8	<0.01	<0.01	⨁◯◯◯ very low
Lipid metabolism-related transcription factors	4	64	−2.36	0.15	98	88.4	<0.01	<0.01	⨁◯◯◯ very low

Significant parameters in bold.

**Table 2:** Subgroup analysis.

Subgroups	SMD	95% CI	*p*	*I*² (%)	95% CI	*p* (subgroup)
Species						0.85
Rat	−2.01	−3.9; −0.1	0.04	89.1	0.8; 0.94	
Mouse	−1.8	−2.8; −0.8	>0.01	82.2	0.69; 0.9	
Intervention duration						0.52
<6 weeks	−2.35	−4.5; −0.17	>0.01	91	0.84; 0.95	
>6 weeks	−1.6	−2.3; −0.9	>0.01	72	0.48; 0.85	
Age						0.15
<8 weeks	−1.02	−1.8; −0.2	0.01	77	0.54; 0.88	
>8 weeks	−3.37	−5.6; −1.2	>0.01	92	0.87; 0.96	
Supplement type						**0.02**
Fish-derived	−2.77	−4.2; −1.4	>0.01	89	0.82; 0.93	
Non-fish-derived	−0.6	−1.3; 0.1	0.07	54	0; 0.83	

*SMD* standardized mean difference. Significant parameters in bold.

### Glucose metabolism

Eight studies involving 120 rodents revealed a significantly alleviated glucose metabolism in the CP group (Fig. 1B; *p* = 0.02; SMD = −2.21; CI = −3.92, −0.49), although potential publication bias (*p* < 0.01, Fig. S3) and heterogeneity (*I*² = 91.6%) were considered high.

### Food intake

Ten trials comprising 192 rodents elicited a significantly reduced food intake in the CP supplementation group (Fig. 1C; *p* = 0.01; SMD = −1.43; CI = −2.56, −0.3), even though heterogeneity was found to be high (*I*² = 88%) and publication bias existed (*p* = 0.02, Fig. S2).

### Adipose tissue and organ content and adipocyte size

**Fig. 2:**
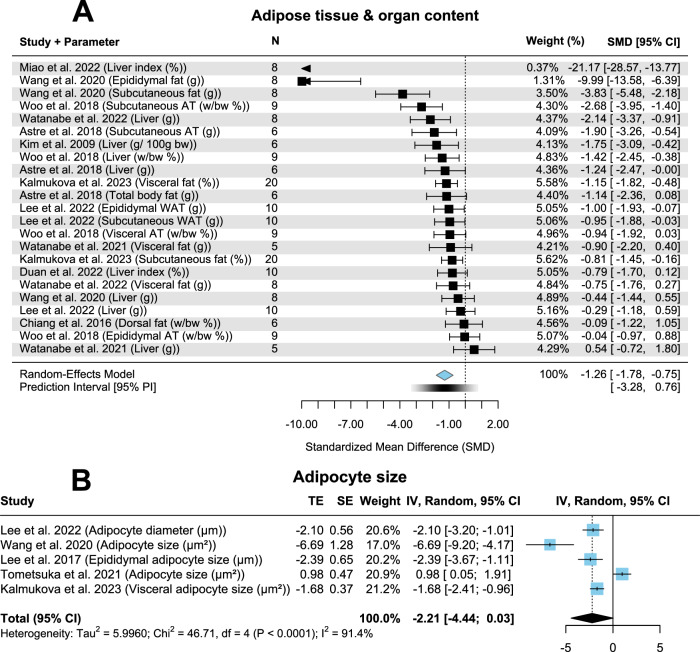
Forest plots showing adipose tissue & organ content as well as adipocyte size with pooled estimates, study weights and confidence intervals. **A** Adipose tissue & organ content. **B** Adipocyte size.

Ten trials including 176 rodents demonstrated a significantly lower amount of adipose tissue and organ content when administering CPs (Fig. 2A; *p* < 0.01; SMD = −1.26; CI = −1.78, −0.75). Publication bias was evident (*p* < 0.01, Fig. S3). Heterogeneity was also given to a high extent (*I*² = 75.2%). Five studies examining adipocyte size in 112 rodents led to a non-significant result between both control and CP group (Fig. 2B; *p* = 0.053; SMD = −2.21; CI = −4.44, 0.03). High heterogeneity (*I*² = 91.4%) and possibly no potential publication bias (*p* = 0.3, Fig. S2) was detected.

### Serum lipids

**Fig. 3:**
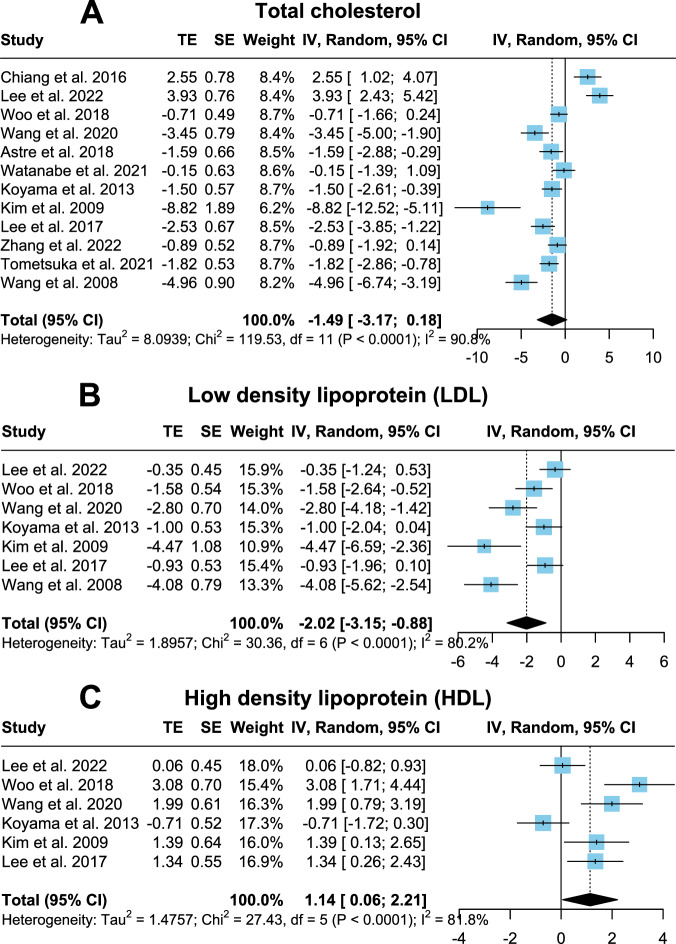
Forest plots showing total cholesterol, LDL and HDL with pooled estimates, study weights and confidence intervals. **A** Total cholesterol. **B** LDL. **C** HDL.

**Fig. 4:**
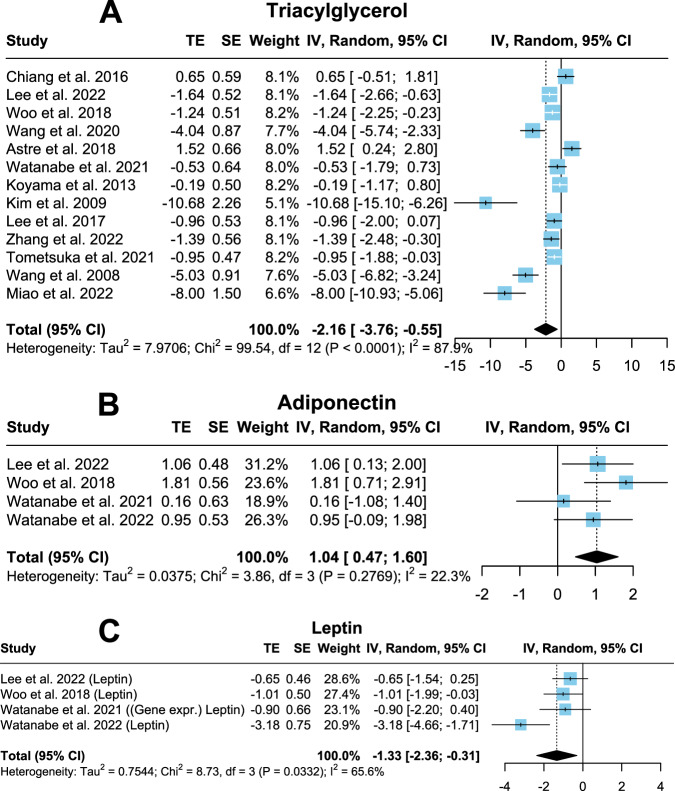
Forest plots showing triacylglycerol, adiponectin and leptin with pooled estimates, study weights and confidence intervals. **A** Triacylglycerol. **B** Adiponectin. **C** Leptin.

Twelve studies comprising 188 rodents investigating total cholesterol reported no significant differences between control and CP group (Fig. 3A; *p* = 0.08; SMD = −1.49; CI = −3.17, 0.18). Heterogeneity remained high (*I*² = 90.8%) and Egger’s regression test did not hint to publication bias (*p* = 0.39, Fig. S2). Seven trials measuring LDL levels in 118 rodents resulted in a significantly lower LDL amount following prolonged CP administration (Fig. 3B; *p* < 0.01; SMD = −2.02; CI = −3.15, −0.88). However, high heterogeneity (*I*² = 80.2%) and publication bias were calculated (*p* = 0.01, Fig. S2). High-density lipoprotein (HDL) gauged in 98 rodents in six studies has been shown to be elevated in CP groups (Fig. 3C, *p* = 0.04; SMD = 1.14; CI = 0.06, 2.21). Nonetheless, high heterogeneity (*I*² = 81.8%) and publication bias were present (*p* = 0.04, Fig. S2). Regarding triacylglycerol (TG), 13 studies involving 204 rodents demonstrated a significantly lower TG in the CP group (Fig. 4A; *p* < 0.01; SMD = −2.16; CI = −3.76, −0.55). Publication bias (*p* < 0.01, Fig. S2) and high heterogeneity (*I*² = 87.9%) existed.

### Adipokines (leptin and adiponectin)

Four studies (64 rodents) investigating adipokines (leptin and adiponectin) showed significant differences following CP supplementation for adiponectin (increased, *p* < 0.01; SMD = 1.04; CI = 0.47, 1.6) and leptin (decreased, *p* = 0.01; SMD = −1.33; CI = −2.36, −0.31; both Fig. 4B, C, respectively). Very low (*I*² = 22.3%) and moderate (*I*² = 65.6%) heterogeneity was calculated for adiponectin and leptin, respectively. Publication bias was not observed in both adiponectin (*p* = 0.6) and leptin (*p* = 0.21, both Fig. S2).

### Oxidative capacity

**Fig. 5:**
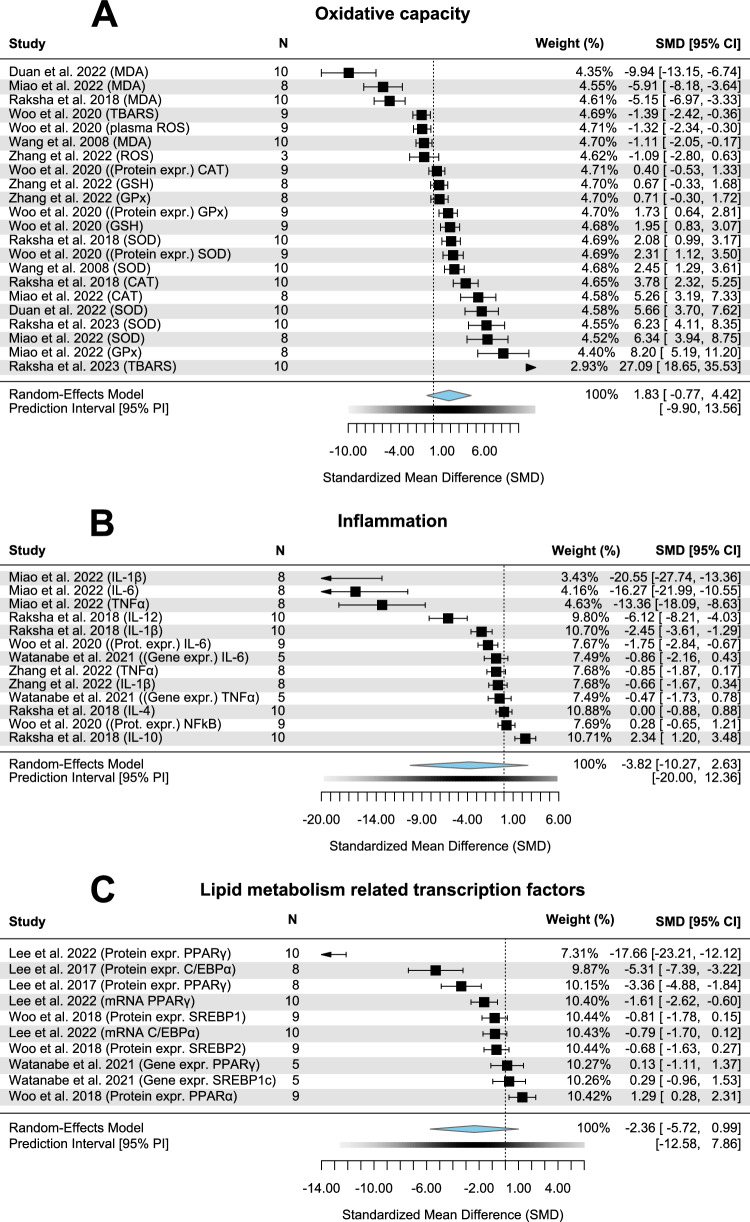
Forest plots showing oxidative capacity, inflammation and lipid metabolism related transcription factors with pooled estimates, study weights and confidence intervals. **A** Oxidative capacity (signs of TBARS, ROS and MDA have been reversed since lower levels exhibit better oxidative protection). **B** Inflammation (signs of IL-4 and IL-10 have been reversed since higher levels exhibit an enhanced anti-inflammatory state). **C** Lipid metabolism related transcription factors (sign of PPARα has been reversed since higher levels exhibit enhanced lipid metabolism).

A non-significantly higher oxidative capacity could be disclosed in the CP group of seven studies, including 120 rodents (Fig. 5A; *p* = 0.16; SMD = 1.83; CI = −0.77, 4.42). A high heterogeneity (*I*² = 98.5%) and no publication bias (*p* = 0.09, Fig. S3) were observed.

### Inflammation

Five studies examining the inflammatory status of 80 rodents illustrated no significantly anti-inflammatory effect of CP administration compared to HFD/ HFC only (Fig. 5B; *p* = 0.22; SMD = −3.82; CI = −10.27, 2.63). Publication bias (*p* < 0.01, Fig. S3) and high heterogeneity (*I*² = 99.1%) were apparent.

### Lipid metabolism-related transcription factors

The pooled expression of specific transcription factor involved in lipid metabolism and energy balancing in four studies comprising 64 rodents were not found to be significantly downregulated when CP was supplemented (Fig. 5C; *p* = 0.15; SMD = 2.36; CI = −5.72, 0.99). Heterogeneity was high (*I*² = 98%) and publication bias evident (*p* < 0.01, Fig. S3).

### Microbiome

The Firmicutes/Bacteroidetes ratio as a descriptor of the gut microbiome was measured in three studies, with one not stating the number of rodents [33]. The results (PLA = 6.75 CP = 1.64 [26], PLA = 10.41 ± 4.01 CP = 5.26 ± 1.33 [33], PLA = 0.57 CP = 1.5 [36]) suggest no clear direction since two studies found higher ratios (both significant) for control groups and one study for the CP group (not significant). A dose-dependent manner was evident for a decreasing F/B ratio with increasing CP dose [26].

### Risk of bias and certainty of evidence

The risk of bias, which was assessed by using the SYRCLE tool [28] that has been developed (and adapted based on Cochranes RoB tool) especially for preclinical animal experiments, can be considered rather high (Table 3). Items 3–7 have been marked as “unclear” in every single study (except Wang et al.^13^, since only the abstract, which included all the data, was available). The corresponding authors did not provide any information (after written request) in the full-text manuscripts regarding allocation concealment, random housing, (investigator/caregiver) blinding, random outcome assessment and blinding of outcome assessor. Most of the trials (*n* = 20) were found to be free of selective outcome reporting and showed groups being similar baseline characteristics (*n* = 17). A method to generate an allocation sequence was only applied and mentioned in half of the trials. With respect to other sources of bias, two studies declared to have been funded by the CP manufacturer. Otherwise, no other sources have been recognized. The certainty of evidence evaluated by means of the GRADE approach appears to be very low overall (Table 1). This is due to high risk of bias, inconsistency and indirectness (due to analysis of animals only) of results, imprecision and possible publication bias (at least for analyses including more than 10 studies).

**Table 3:** Risk of bias assessment by means of the SYRCLE tool.

Study	1	2	3	4	5	6	7	8	9	10
Chiang et al. 2016	U	Y	U	U	U	U	U	U	Y	Y
Raksha et al. 2023	Y	U	U	U	U	U	U	Y	Y	Y
Lee et al. 2022	N	Y	U	U	U	U	U	U	Y	N
Woo et al. 2018	N	Y	U	U	U	U	U	Y	Y	Y
Baek et al. 2023	Y	Y	U	U	U	U	U	U	Y	Y
Wang et al. 2020	U	Y	U	U	U	U	U	Y	Y	Y
Astre et al. 2018	U	Y	U	U	U	U	U	Y	Y	Y
Watanabe et al. 2021	U	Y	U	U	U	U	U	Y	N	Y
Koyama et al. 2013	N	Y	U	U	U	U	U	U	Y	U
Woo et al. 2020	N	Y	U	U	U	U	U	Y	Y	Y
Kim et al. 2009	Y	Y	U	U	U	U	U	Y	Y	U
Duan et al. 2022	Y	Y	U	U	U	U	U	U	Y	Y
Lee et al. 2017	Y	Y	U	U	U	U	U	N	Y	Y
Zhang et al 2022	Y	U	U	U	U	U	U	Y	Y	Y
Tometsuka et al. 2021	N	U	U	U	U	U	U	U	Y	Y
Kalmukova et al. 2023	Y	U	U	U	U	U	U	U	Y	Y
Guo et al. 2022	Y	Y	U	U	U	U	U	U	Y	Y
Watanabe et al. 2022	N	Y	U	U	U	U	U	U	Y	N
Raksha et al. 2018	Y	Y	U	U	U	U	U	Y	Y	Y
Miao et al 2022	Y	Y	U	U	U	U	U	Y	Y	Y
Wang et al. 2008 (only abstract)	-	-	-	-	-	-	-	-	-	-

*Y* yes, *U* unclear, *N* no.

## Discussion

To our knowledge, this is the first systematic review and meta-analysis to investigate the effects of long-term CP administration in rodents on a high-fat or HCD. The findings suggest that CP supplementation appears to significantly decrease body mass, food intake, leptin, adipose tissue content, LDL and TG. Enhanced adiponectin levels and attenuated glucose metabolism have been observed. However, the overall certainty of evidence seemed very low.

### Effects on body mass, food intake, leptin and adipose tissue

The results of the current meta-analysis show CPs superiority in decreasing rodent body weight, food intake and adipose tissue. A several-week administration of sole glycine, the most abundant amino acid in CPs, even showed a pronounced loss of whole-body fat and epididymal fat while preserving lean mass and quadriceps muscle mass in mice facing obesity undergoing caloric restriction [37]. Glycine intake in mice has also been reported to prevent maturity-onset obesity and hepatic steatosis [38], a disease elicited by fatty acid and TG accumulation within the liver that might ultimately result in cirrhosis and even hepatocellular carcinoma [39]. Glycine also facilitates excitatory transmission in the brain via activation of the N-methyl-d-aspartate receptor [40], which seems to be involved in the regulation of food intake [41]. In addition, glycine receptor (GlyR) protein levels were augmented in the hypothalamus of HFD-fed mice, implicating a potential role of GlyR in obesity-related orexogenic signaling [42]. Therefore, glycine as a primary source of CP could also be of interest for human clinical testing in the context of obesity [43].

Furthermore, an in-vitro trial delivered evidence of hydrolyzed collagen (HC) conjugated with epigallocatechin gallate inhibiting the activity of obesity associated enzymes α-amylase and α-glucosidase [44], but with reduced inhibitory potential when HC was applied alone. In human exercise trials lasting several months where subjects typically exercise three times a week, daily CP administration has significantly augmented lean body mass [45], evidenced by numerous experiments, even while reducing fat mass (FM) [46, 47]. Therefore, CP supplementation could be a beneficial adjunct for patients with obesity as well, and in fact, a recent study has demonstrated a significantly reduced FM following a 12-week CP regimen compared to a placebo in middle-aged, untrained men with obesity [48]. Body weight reductions alongside decreased body FM [49] have been reported following 12-weeks of daily CP intake in adults ranging from normal weight to overweight [50] without concomitant significant declines in food intake [51] expressed as total kcal consumed. In another human randomized controlled trial, individuals with obesity who did not receive a prescribed exercise intervention were administered bovine collagen—technologically modified to enhance its water-binding capacity—over a period of 12 weeks. Although energy and macronutrient intake did not differ significantly between groups after the intervention, the collagen group experienced significantly greater weight loss (~1.5 kg), increased satiety and reduced hunger (both assessed by questionnaire data). Additionally, in a preceding experiment, postprandial blood ghrelin levels of rats were also significantly lower following collagen intake compared to casein [52]. Together with the aforementioned findings, this highlights collagen protein’s potential to promote satiety through its distinct physical properties in humans. However, it remains to be elucidated whether effects on orexigenic hormones also occur in humans.

Leptin, a peptide hormone mainly produced in adipose tissue and involved in the regulation of body mass and food intake, was significantly reduced by CP intake in the present meta-analysis. This is in line with trials including subjects with obesity [53] and massive obesity [54] (BMI of 35 and above), illustrating higher serum leptin and mRNA expression levels (ex vivo) than normal-weight controls and a strong positive association between blood leptin concentrations and body fat percentage. The mRNA content of the ob gene (responsible for leptin production) was also twice as high in the group with obesity, suggesting subjects with obesity have developed leptin resistance that might be the consequence of lowered leptin receptor expression or disturbed leptin receptor signaling [55]. Since mechanisms regarding leptin and CPs are currently unknown, we suppose that CP-induced reductions in weight and food intake might have caused concomitant low leptin levels, at least in rodents. Moreover, the significant decrease in adipose tissue and organ content in the current meta-analysis may have contributed to low leptin levels since adipose tissue is majorly involved in leptin production. Furthermore, adipose tissue organ content and adipocyte diameter or cross-sectional area, usually gauged ex vivo, are often not investigated in human trials, which dampens the transferability of potential CP effects in humans. Apart from that, mixed results have been reported from two long-term human trials supplementing CPs for 10–12 weeks in individuals with a BMI above 25. In one study, leptin levels decreased in both groups but remained significantly higher in the CP group compared to placebo [52]; in the other, leptin levels remained stable, with a tendency to increase in the whey protein group compared to CP [56]. Hence, robust human clinical evidence is still lacking regarding the effects of CPs on leptin regulation and related metabolic outcomes.

### Effects on serum lipids and glucose metabolism

The current meta-analysis revealed significant reductions in serum lipid markers (LDL and TG). Additionally, HDL levels increased, while total cholesterol levels remained largely unchanged under the caveat of high heterogeneity. One underlying mechanism appears to be CPs potential influence on lipid absorption, which may be effective in suppressing transient blood TG increases as demonstrated in rats [57]. Plasma TG concentration has been reported to negatively correlate (*r* = 0.53–0.63) with plasma glycine, proline and hydroxyproline, highlighting fish-derived CP amino acids’ ability to counteract TG accumulation [57]. Fish CPs, especially Hyp- comprising peptides, have also been found in higher abundance in human blood compared to porcine-derived CPs [58]. As higher levels of blood CPs might inhibit TG absorption, low-molecular-weight (to enhance absorption) fish-specific CP administration could provide lipid-lowering effects in obesity. As certain lipid markers have also been improved in the current review of preclinical studies, such findings have yet to be observed in human trials [49–51], particularly those involving subjects with obesity [56], since dyslipidemia displays a crucial risk factor for developing cardiovascular diseases and is even associated with non-alcoholic fatty liver disease and acute pancreatitis [59].

Alleviated glucose metabolism (expressed as glucose, insulin and glycated hemoglobin (HbA1c)) was also observed in the present meta-analysis. High-dose CPs (1.7 g/kg bodyweight) derived from tilapia skin exhibited similar glucose-lowering effects evoked by metformin in diabetic mice after 25 days [60]. CPs from harpadon nehereus bones elicited favorable up- and downregulations of certain key enzymes following a 4-week administration in diabetes type 1 (DMT1) mouse models. The CP-induced expression levels of glucose-6-phosphatase (G6Pase) and phosphoenolpyruvate carboxykinase 1 (PEPCK1) in the liver of the diabetic mice were reduced, while the Glucokinase (GK) and phosphorylation of Glycogen synthase kinase-3 (GSK-3β) were elevated. In addition, apoptosis in pancreatic cells and hepatocytes as well as cell swelling and alanine transaminase (ALT) & aspartate aminotransferase (AST) could be lowered, which might indicate CPs to ameliorate DMT1-induced liver damage [61]. In a human trial, 5 g of fish CPs were also able to improve resting blood glucose and insulin sensitivity in adults with DMT2 following a 90-day supplementation phase [62]. These results, plus reduced HbA1c levels, were also obtained in Chinese adults with DMT2 and normal weight after 6 and 9 months of daily 13 g marine CP supplementation [63]. At least in patients with diabetes, CPs from sheep skin have been shown to inhibit dipeptidyl peptidase-IV (DPP-IV) activity [64] that prolongs and increases the activity of the incretin hormones glucagon-like peptide-1 and glucose-dependent insulinotropic polypeptide, which act as key prandial stimulators of insulin secretion and regulators of blood glucose control [65]. Fish-derived CPs might thus be a suitable supplement for individuals with DMT2 [66] and probably for patients with obesity and dysregulated glucose metabolism. Nevertheless, glucose metabolism has not been influenced by an 8-week CP administration regimen in adults with overweight, demonstrated by unchanged levels of glucose, insulin and the homeostatic model assessment of insulin resistance (HOMA-IR) and β cell function (HOMA-β; both being markers of insulin dynamics [56, 67]).

### Effects on inflammation, oxidative stress, and adiponectin

Somewhat surprisingly, significant anti-inflammatory effects as well as enhanced oxidative capacity following CP administration have not been observed in the present meta-analysis. In a mouse model treated with D-galactose together with UV irradiation, a 7-week chicken bone-derived CP administration inhibited skin inflammation and improved levels of antioxidants. Moreover, lysosome-related genes were upregulated, indicating enhanced degradation of cellular debris/protein aggregates [68]. Other than the investigated inflammatory and oxidative parameters in the current review, an in-vitro experiment referred to CPs (extracted from chicken sternal cartilage) ability to scavenge ABTS and DPPH radical (two common assays used for measuring radical scavenging) and alleviating H_2_O_2_-induced cellular oxidative damage in rat knee joints, probably caused by low-molecular-weight and hydrophobic & antioxidant amino acid residues. Pro-inflammatory cytokine secretion was also decreased, probably elicited from increased synthesis of extracellular matrix key components combined with suppressed chondrocyte apoptosis [69]. Milkfish CPs also revealed good ABTS and DPPH radical scavenging as well as reductions in lipoxygenase activity, nitric oxide radicals, and DNA single-strand breaks in vitro [23]. A recent advance by using ultrasound-assisted (450 W) enzymatic hydrolysis for CP preparation demonstrated enhanced peptide and α-helix content together with decreased random coil and β-chain, thus likely acting anti-inflammatory in lipopolysaccharide (LPS)-induced cells [70]. LPS- induced inflammation was also mitigated in mice by Tilapia-derived CPs [71]. Inflammation specifically taking place in the colon of ulcerative colitis-positive mice has been ameliorated by CP-provoked upregulation of mitogen-activated protein kinase phosphatase-1 (MKP-1) and by that reducing the phosphorylation levels of both signaling proteins c-Jun N-terminal kinases (JNK) and p38 mitogen-activated protein kinases (P38), finally attenuating pro-inflammatory cytokine expression (TNF-α, IL-1β, and IL-6) [72]. A recent investigation in Dextran Sodium Sulfate induced colitis mice reported fish CPs acting protectively against colitis by directly influencing macrophages, steering their polarization towards an anti-inflammatory, immunotolerant, and antioxidant phenotype in a mannose receptor-dependent manner. Additionally, CPs impact on the immune system may help sustain intestinal eubiosis [73]. Another approach in attenuating inflammation seems to be targeting the nuclear factor kappa-B (NF-κB) signaling pathway. In acute kidney injury mice [74] and human HaCaT keratinocyte cells [75], CPs diminished NF-κB along with other pro-inflammatory cytokines. Furthermore, six peptides (GPAGPSGPAGK, GPAGPSGPAGKDGR, GPSGPQGIR, GPAGPQGPR, GEAGPAGPAGPAGPR, and GEGGPQGPR) have been shown to regulate NF-κB signaling pathways together with nitric oxide production to act anti-inflammatorily [76]. Nonetheless, human trials on CP supplementation have not yet reported significant anti-inflammatory effects, at least following muscle damage-induced inflammation [77, 78]. Two 24-week multicenter trials in patients with rheumatoid arthritis—a chronic autoimmune and inflammatory joint disease [79]—reported improved joint function and enhanced therapeutic efficacy following supplementation with CP (derived from type II collagen). These effects were observed even at low doses of 20 µg [80] and 100 µg/d [81]. Although CRP levels did not decrease in one of the trials, the results provide preliminary indirect evidence for the anti-inflammatory potential of CPs in humans.

Adiponectin, a hormone synthesized in white adipose tissue, has been found significantly elevated after CP administration. Glycine, as the major amino acid in collagen, has been reported to significantly enhance adipocyte-released adiponectin mRNA in a 3T3-L1 cell line [82]. Adiponectin has already been reported to improve insulin sensitivity, enhance fatty acid transportation, provide anti-atherosclerosis effects and even modulate inflammatory responses by means of targeting mast cells, eosinophils and macrophages [83, 84]. Its anti-inflammatory properties might derive from its ability to lower CRP and its corresponding mRNA, inhibiting NF-κB signaling and macrophage-specific secretion of TNFα [85]. Ostensibly, mainly glycine from CPs might foster these processes as this collagen-specific amino acid has also demonstrated enhanced mRNA levels of adiponectin and IL-10 without affecting adipogenesis in vitro [86], suppression of pro-inflammatory cytokines production and increasing adiponectin secretion in vivo via activation of PPARγ in mice [87]. In a human cross-sectional study [88], a negative correlation has been found between adiponectin and fasting plasma glucose in metabolically healthy individuals with obesity (those without metabolic syndrome). However, a recent review highlighted paradoxical effects of both adipokines, suggesting that high or low levels may not actually confer beneficial effects on cardiovascular function [89], which is why therapeutic agents might seek a slight adaptation of these cytokines. To our knowledge, just a single study has investigated CPs impact on adipokines in women with overweight following an 8-week administration regimen, resulting in unchanged leptin and adiponectin levels (accompanied with stabilized lean body mass) [56]. CPs mechanistic influence on adiponectin and vice versa and their role in obesity is still not fully understood.

### Effects on transcription factors involved in lipid metabolism

Lipid metabolism-related transcription factors have not revealed a significant decrease in the present systematic review. The peroxisome proliferator-activated receptor (PPAR) family are widely known as metabolic regulators, modulating lipid homeostasis. PPARα & γ, which were included in the meta-analysis, are mainly responsible for fatty acid β-oxidation and the storage of TGs in adipocytes, respectively [90]. PPARγ, together with CCAAT/enhancer binding protein α (C/EBPα) mRNA and protein expression, another key transcription factor in adipogenesis [91], could be downregulated in the studies included in the current analysis. PPARα upregulation was also achieved with CPs in the liver of db/db mice alongside a downregulation of SREBP-1 & 2, indicating a substantial suppression of hepatic lipid accumulation [92]. An animal investigation of glycine administration alone resulted in a reduction of PPARγ mRNA expression in the liver after 4 and 8 weeks, but not in adipose tissue of monosodium glutamate-induced mice with obesity (MSG/Ob mice) compared to lean ones [93]. Surprisingly, liver PPARα mRNA expression significantly decreased in these MSG/Ob mice, indicating that the anti-obesity effects of CPs may be attributed to amino acids or peptides other than glycine. Prolyl-hydroxyproline (Pro-Hyp), a bioactive peptide abundantly found in CPs, has recently been reported to decrease adipocyte size, to increase mitochondrial activity and to upregulate brown fat-specific genes such as C/EBPα and PPARγ coactivator-1 alpha (PGC-1α) without altering PPARγ expression. Surprisingly, a Pro-Hyp-responsive element was identified in the PGC-1α gene promoter, enabling Foxg1 (= transcription factor) to bind and increase PGC-1α expression. This, in turn, promotes brown adipocyte differentiation, highlighting a potential anti-obesity effect—at least in an in vitro setting [94]. Currently, it remains unknown whether CPs or collagen-specific amino acids exert any influence on lipid metabolism-related transcription factors in humans.

### Effects on the gut microbiome

Microbiome analysis revealed equivocal findings in this systematic review, exhibiting lower Firmicutes/Bacteroidetes ratios after CP intake in two out of three studies. A high-fat diet commonly leads to a Firmicutes (Clostridium) dominant gut microbiota, while being deficient in beneficial genera/species such as Bacteroides, Bifidobacterium or Lactobacillus. This altered profile of the gut is associated with decreased short-chain fatty acids, which are essential for maintaining intestinal epithelial barrier integrity, reducing inflammation and bacterial translocation, and increasing hunger-suppressing hormone expression [95]. In an iron-deficiency anemia rat model, inducing high abundances of Firmicutes, a chelate of pig skin CP and Fe^2+^ supplemented for 3 weeks, conveyed a higher number of Bacteroidetes and fixed the iron-deficiency anemia evoked decrease in the relative abundances of Firmicutes and Bacteroidetes [96]. A downgrade of Subdoligranulum relative abundance, a bacteria that might be relevant for mitigating DMT2 and obesity [97], was also observed with CP intake in this experiment. To the best of our knowledge, only a single trial has examined the impact of a 2-week CP intervention on the gut microbiota, specifically in patients suffering from major burn injuries [98]. Bifidobacterium levels significantly decreased in both the placebo and collagen + sunflower oil groups, whereas no change was observed in the collagen + fish oil group. Notably, the Firmicutes/Bacteroidetes ratio remained stable only in the collagen + fish oil group, likely due to its high omega-3 content. However, further human trials investigating potential microbiota changes following prolonged CP supplementation are still lacking, leaving the effects on the human microbiome and obesity largely uncertain.

### Limitations

As preclinical animal studies differ in several aspects (e.g., species, design, age, etc. [99]) from clinical human trials, mostly high heterogeneity was observed throughout the current meta-analysis. The subgroup analysis undertaken for one parameter having the highest number of studies included revealed that disparities in intervention length and CP source existed, which might have caused high between-study variability and total variation. An overall poor methodological quality was present, which substantially enlarged the risk of bias. Moreover, many authors have not responded to our written requests, which is why a lot of data had to be extracted with the web plot digitizer tool, a procedure that might have also elicited some variability. The certainty of evidence assessed by the GRADE approach remained very low for each parameter due to inconsistency, indirectness and risk of bias. Recent research on animal-to-human translation reported a median success rate of approximately 65%, with a range from 0 to 100% [100]. In light of the aforementioned limitations, translating the present findings to humans remains particularly challenging [101]. However, the doses applied in the analyzed trials ranged from 0.01 to 4.5 g/kg body weight in rodents, corresponding to a maximum human equivalent dose of approximately 51 g for a 70 kg individual [102]—a dose that has already been administered in human clinical trials [103, 104]. Lastly, the CPs included in the present review were extracted from various animal sources, each potentially exerting distinct effects [105]. The identification of novel di-, tri-, and oligopeptides with efficacy against metabolic disorders in humans remains an important subject for future research.

## Conclusion

CP administration in rodent obesity models of at least 3 weeks demonstrated significant beneficial effects with respect to body mass, food intake, adipose tissue content, endocrine functioning, adipokines, glucose metabolism, and lipid serum markers. More research is definitely needed in the field of CP-related anti-obesity effects in human trials, given that a handful of studies majorly examined blood lipids and glucose together with body composition (body weight, fat & fat-free mass) variables. Although not significantly enhanced in the current meta-analysis (which included only four studies), CPs may influence lipid metabolism through the molecular regulation of specific transcription factors. Future studies are encouraged to further investigate the molecular mechanisms of CP supplementation in humans. Future preclinical trials should highly consider randomization of animals and blinding of researchers and following the ARRIVE guidelines 2.0 [106] to improve overall reporting quality, since reporting prevalence is still lacking in experimental animal research [107].

## Supplementary information

Supplementary file 1

## Data Availability

The datasets generated during the current systematic review and meta-analysis are available from the corresponding author on reasonable request.
