# Supplementary material for: Regular collagen peptide administration exerts anti-obesity effects in high-caloric diet-fed rodents—a systematic review with meta-analysis of animal trials
Source: Int J Obes (Lond). 2025 Oct 30;50(1):8–22. doi: 10.1038/s41366-025-01905-3 (PMC12855012; doi:10.1038/s41366-025-01905-3)
Supplement: Supplementary file 1 [file 41366_2025_1905_MOESM1_ESM.pdf]

Supplementary file 1

(includes Figure S1, S2, S3 and Table S1)

Regular collagen peptide administration exerts anti-obesity effects in high-caloric diet-fed rodents – A systematic review with meta-analysis of animal trials

Kevin Bischof [2,3*], Anna Maria Moitzi [1,3], Daniel König [1,2,3]

[1] Faculty of Life Sciences, Department for Nutrition, Section for Nutrition, Exercise and Health, University of Vienna, Vienna, Austria

[2] Centre for Sports Science and University Sports, Department of Sports Science, Section for Nutrition, Exercise and Health, University of Vienna, Vienna, Austria

[3] Vienna Doctoral School of Pharmaceutical, Nutritional and Sport Sciences, University of Vienna, Vienna, Austria

*** Correspondence:**
Kevin Bischof, https://orcid.org/0009-0008-6416-6524
kevin.bischof@univie.ac.at

Figure S1: PRISMA flow chart of analyzed studies

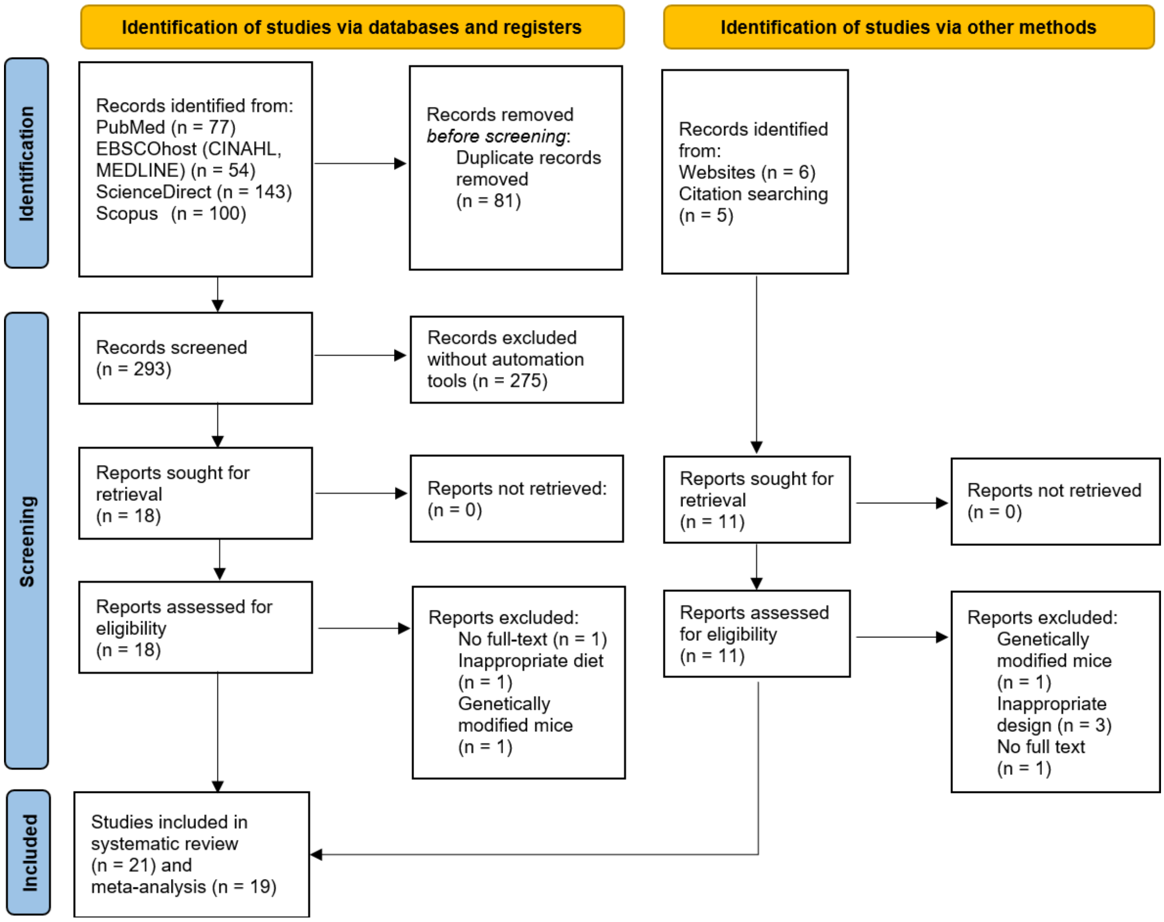

Figure S2: Funnel plots of body mass, food intake, adipocyte size, total cholesterol, HDL, LDL, adiponectin, leptin and triacylglycerol. Derived from conventional meta-analysis. X-axis depicts standardized mean difference (Hedges' g).

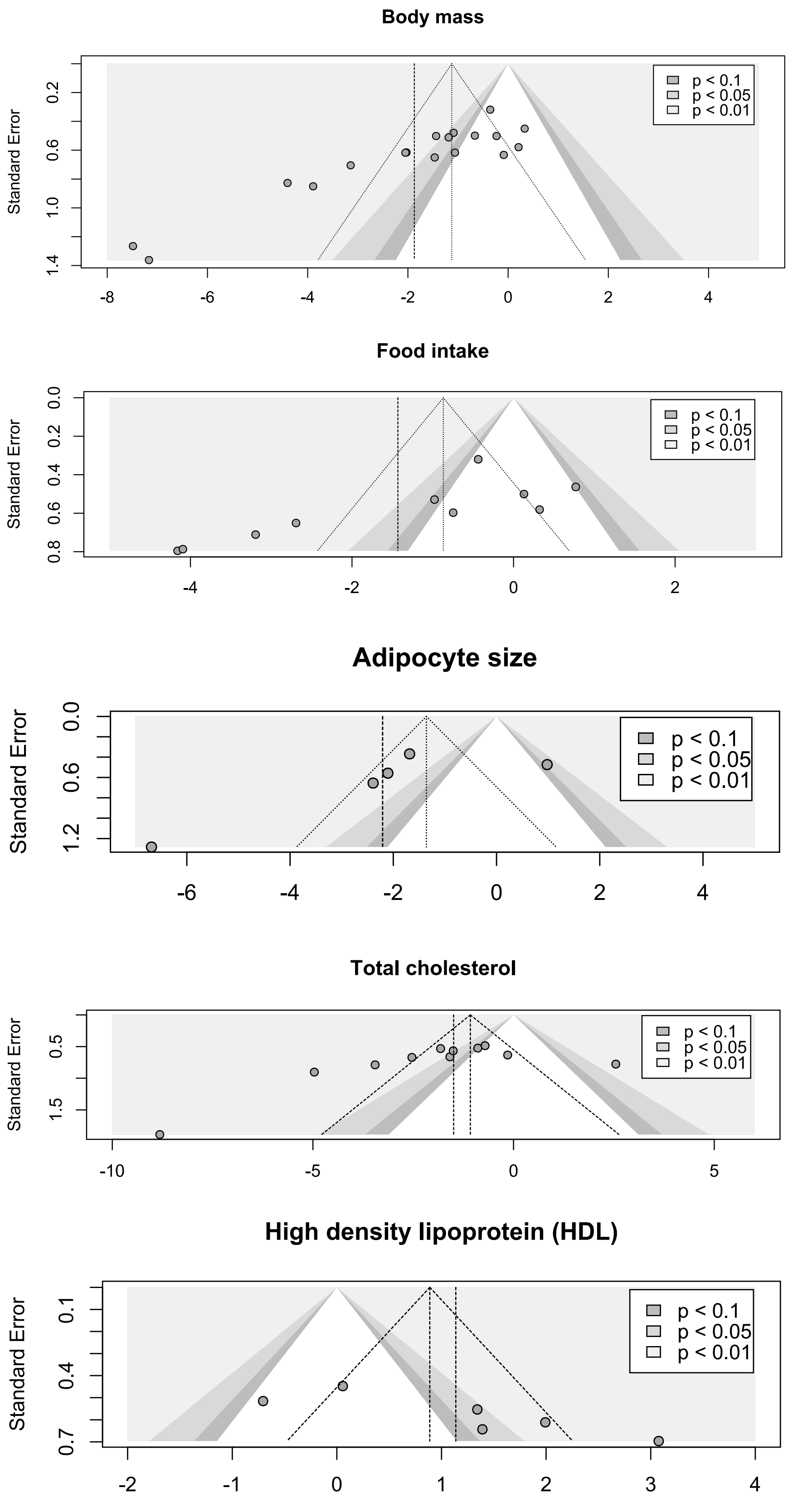

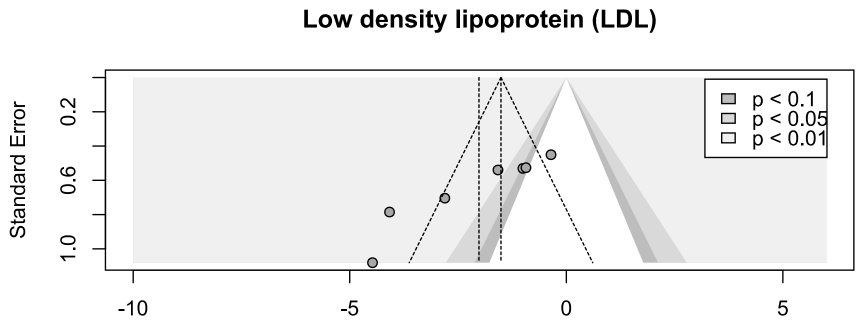
Low density lipoprotein (LDL)
Standard Error
0.2
0.6
1.0
-10
-5
0
5
p < 0.1
p < 0.05
p < 0.01

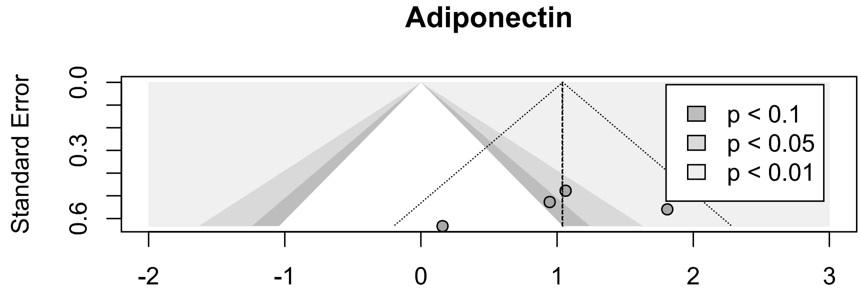
Adiponectin
Standard Error
0.0
0.3
0.6
-2
-1
0
1
2
3
p < 0.1
p < 0.05
p < 0.01

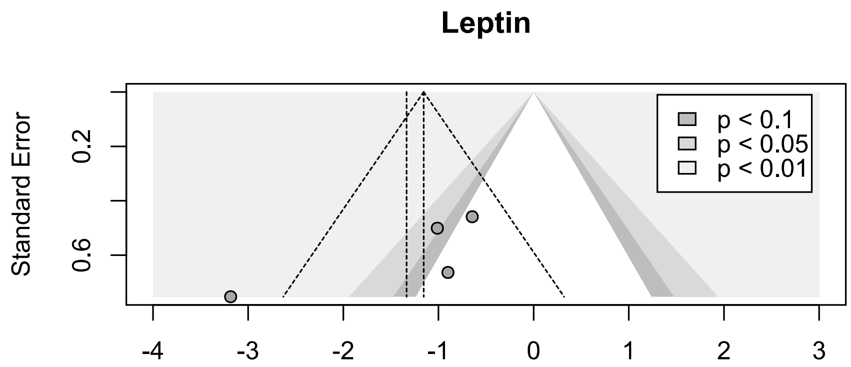
Leptin
Standard Error
0.2
0.6
-4
-3
-2
-1
0
1
2
3
p < 0.1
p < 0.05
p < 0.01

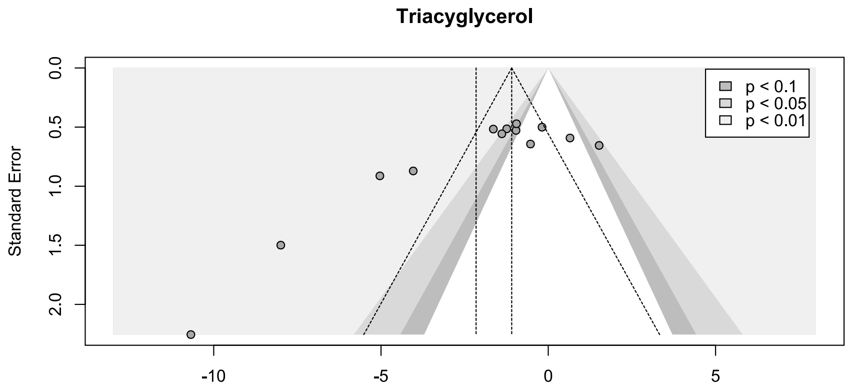
Triacyglycerol
Standard Error
0.0
0.5
1.0
1.5
2.0
-10
-5
0
5
p < 0.1
p < 0.05
p < 0.01

Figure S3: Funnel plots of glucose metabolism, adipose tissue & organ content, oxidative capacity, lipid metabolism related transcription factors and inflammation. Derived from multilevel meta-analysis. X-axis depicts standardized mean difference (Hedges' g).

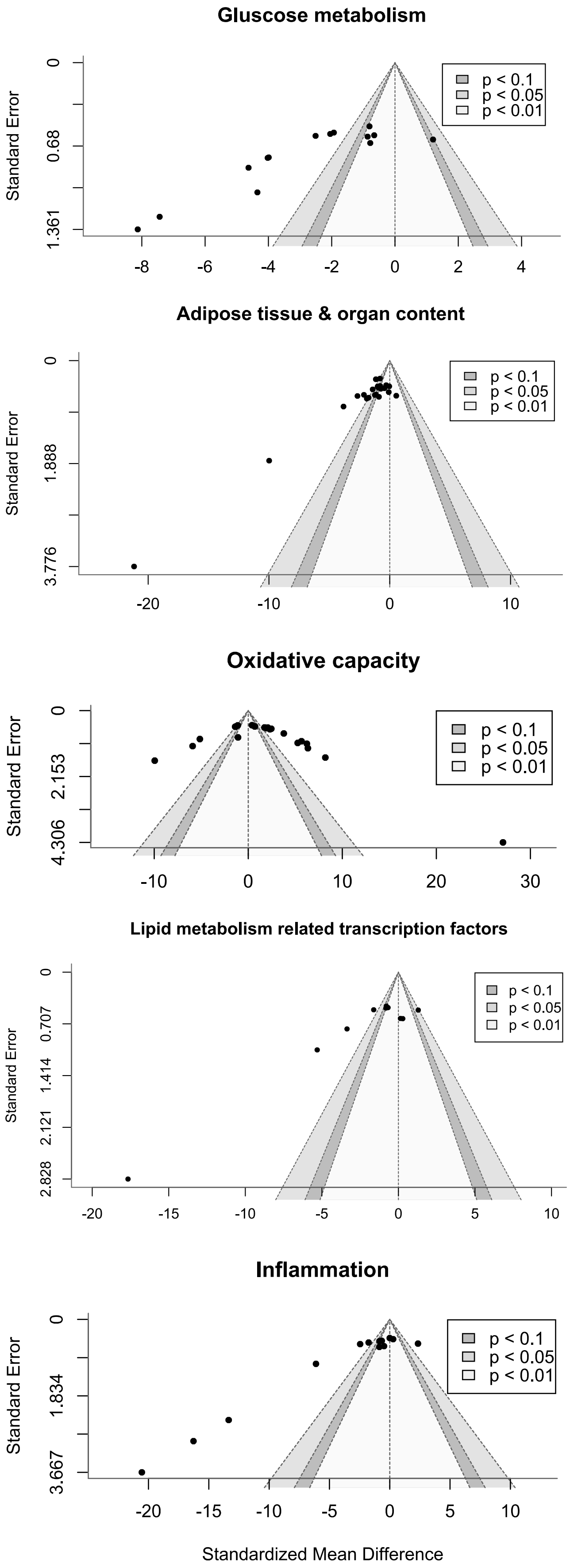

Table S1: Characteristics of included studies in the systematic review and meta-analysis.

Study	n/ group	Design	Strain	Age	Dose	Duration	Placebo diet	Collagen peptides	Outcome
**Chiang et al. 2016**	6	PGD	Sprague Dawley rats	n/a	1.25/ 2.5 mg/ml	12w	n/a	n/a	Body weight (g)<br>PLA= 391.48 ± 16.91<br>CP(1.25)= 389.86 ± 16.25<br>CP(2.5)= 367.75 ± 12.68<br>Dorsal fat (w/bw %)<br>PLA= 0.5 ± 0.13<br>CP(1.25)= 0.44 ± 0.02<br>CP(2.5)= 0.49 ± 0.08<br>Glucose (mg/dL)<br>PLA= 82.56 ± 7.81<br>CP(1.25)= 80.01 ± 6.46<br>CP(2.5)= 97 ± 13.59<br>Cholesterol (mg/dL)<br>PLA= 55.05 ± 6.34<br>CP(1.25)= 59.35 ± 9.41<br>CP(2.5)= 69.78 ± 4.09<br>Triacylglycerol (mg/dL)<br>PLA= 89.78 ± 22.85<br>CP(1.25)= 58.4 ± 6.35<br>CP(2.5)= 107.74 ± 27.93
**Raksha et al. 2023**	10	RCT	Wistar rats	10w	1 g/kg	6w	HCD	Jellyfish (Diplulmaris Antarctica)	ΔBody weight (%)<br>PLA= 165.5 ± 8<br>CP= 132 ± 6.5<br>Food intake (g/d)<br>PLA= 30.5 ± 2.4<br>CP= 20.5 ± 2.2<br>HbA1c (μmol fructose/g hemoglobin)<br>PLA= 0.85 ± 0.06<br>CP= 0.60 ± 0.06<br>Insulin (relative units)<br>PLA= 0.21 ± 0.01<br>CP= 0.14 ± 0.006<br>Glucose (mmol/L)<br>PLA= 7.66 ± 0.50<br>CP= 6.35 ± 0.50<br>SOD(U/mg protein/min)<br>PLA= 2.05 ± 0.41<br>CP= 4.75 ± 0.42<br>TBARS(nmol/mg protein)<br>PLA= 0.71 ± 0.003<br>CP= 0.81 ± 0.004
**Lee et al. 2022**	10	PGD	C57BL/ 6J mice	6w	1.5/4.5 g/kg	12w	HFD	Porcine	Body weight (g)<br>PLA= 38.52 ± 4.55<br>CP(1.5)= 38.36 ± 2.91<br>CP(4.5)= 33.61 ± 4.08<br>Adipocyte diameter (μm)<br>PLA= 103.65 ± 28.93<br>CP(1.5)= 88.35 ± 36.05<br>CP(4.5)= 56.1 ± 9.96<br>Subcutaneous WAT(g)<br>PLA= 1.65 ± 0.73 CP(1.5)=1.51 ± 0.6<br>CP(4.5)=1.05 ± 0.44<br>Epididymal WAT(g)

									PLA=2.22 ± 0.7 CP(1.5)=2.16 ± 0.82<br>CP(4.5)=1.51 ± 0.66<br>Liver(g)<br>PLA= 1.06 ± 0.19<br>CP(1.5)= 1.09 ± 0.13<br>CP(4.5)=1.01 ± 0.13<br>Cholesterol (mg/dL)<br>PLA= 93 ± 6.96<br>CP(1.5)= 131.22 ± 11.73<br>CP(4.5)= 137.1 ± 13.53<br>LDL (mg/dL)<br>PLA= 100.13 ± 23.97<br>CP(1.5)= 87.84 ± 9.77<br>CP(4.5)= 93.3 ± 10.59<br>HDL (mg/dL)<br>PLA= 24.33 ± 4.93<br>CP(1.5)= 23.44 ± 2.66<br>CP(4.5)= 24.6 ± 4.14<br>Triacylglycerol (mg/dL)<br>PLA= 134.7 ± 23.65<br>CP(1.5)= 99.67 ± 14.96<br>CP(4.5)= 96 ± 21.41<br>Adiponectin (ng/mL)<br>PLA= 4.27 ± 0.76<br>CP(1.5)= 4.56 ± 0.73<br>CP(4.5)= 5.13 ± 0.79<br>Leptin (pg/mL)<br>PLA= 2.11 ± 1.11<br>CP(1.5)= 1.8 ± 0.56<br>CP(4.5)= 1.31 ± 1.26<br>(mRNA) PPARγ (% of GAPDH)<br>PLA= 1.81 ± 0.57<br>CP(1.5)= 0.91 ± 0.47<br>CP(4.5)= 0.85 ± 0.57<br>(mRNA) C/EBPα (% of GAPDH)<br>PLA= 3.78 ± 3.23<br>CP(1.5)= 1.29 ± 3.32<br>CP(4.5)= 1.13 ± 3.16<br>(Protein expr.) PPARγ (% of NC)<br>PLA= 6.86 ± 0.44<br>CP(1.5)= 3.03 ± 0.51<br>CP(4.5)= 0.26 ± 0.25
**Woo et al. 2018**	9	PGD	C57BL/ 6J mice	6w	0.1/0.2/ 0.3 g/kg	8w	HFD	Skate (Raja kenojei) skin	Body weight (g)<br>PLA= 36.6 ± 1<br>CP(0.1)= 34.2 ± 0.8<br>CP(0.2)= 33.6 ± 1.1<br>CP(0.3)= 33.3 ± 1<br>Food intake (g/d)<br>PLA= 2.07 ± 0.02<br>CP(0.1)= 1.93 ± 0.11<br>CP(0.2)= 2.01 ± 0.04<br>CP(0.3)= 1.92 ± 0.06<br>Visceral adipose tissue (w/bw %)<br>PLA= 11.11 ± 3.95<br>CP(0.1)= 9.21 ± 1.9<br>CP(0.2)= 8.58 ± 0.91<br>CP(0.3)= 8.26 ± 0.95<br>Subcutaneous adipose tissue (w/bw %)<br>PLA= 2.79 ± 0.47

CP(0.1)= 1.96 ± 0.63
CP(0.2)= 1.85 ± 0.44
CP(0.3)= 1.79 ± 0.18
Epididymal adipose tissue (w/bw %)
PLA= 6.49 ± 1.84
CP(0.1)= 6.49 ± 1.25
CP(0.2)= 6.49 ± 2.35
CP(0.3)= 6.42 ± 1.2
Liver (w/bw %)
PLA= 4.32 ± 0.38
CP(0.1)= 3.97 ± 0.2
CP(0.2)= 3.92 ± 0.17
CP(0.3)= 3.83 ± 0.27
Cholesterol (mg/dL)
PLA= 40.53 ± 3.95
CP(0.1)= 40 ± 2.76
CP(0.2)= 39.21 ± 4.34
CP(0.3)= 38.03 ± 2.63
HDL (mg/dL)
PLA= 3.5 ± 1.55
CP(0.1)= 8.86 ± 2.09
CP(0.2)= 9.77 ± 2.32
CP(0.3)= 11.5 ± 3.14
LDL (mg(dL)
PLA= 17.52 ± 3.42
CP(0.1)= 16.58 ± 3.42
CP(0.2)= 13.89 ± 5.37
CP(0.3)= 10.13 ± 5.3
Triacyglycerol (mg/dL)
PLA= 114.14 ± 32.41
CP(0.1)= 90.34 ± 29.31
CP(0.2)= 80 ± 21.38
CP(0.3)= 80 ± 17.93
Adiponectin (unit n/a)
PLA= 214.4 ± 46.3
CP(0.1)= 236.3 ± 21.5
CP(0.2)= 263.8 ± 35.3
CP(0.3)= 281.1 ± 17.9
Leptin (unit n/a)
PLA= 122.6 ± 34.9
CP(0.1)= 98 ± 24.6
CP(0.2)= 97 ± 22.9
CP(0.3)= 94 ± 15.2
(Protein expr.) PPARα (% of NC)
PLA= 0.6 ± 0.27
CP(0.1)= 0.84 ± 0.2
CP(0.2)= 0.98 ± 0.28
CP(0.3)= 1.01 ± 0.33
(Protein expr.) SREBP1 (% of NC)
PLA= 1.7 ± 0.68
CP(0.1)= 1.66 ± 0.51
CP(0.2)= 1.33 ± 0.34
CP(0.3)= 1.24 ± 0.34
(Protein expr.) SREBP2 (% of NC)
PLA= 1.4 ± 0.3
CP(0.1)= 1.5 ± 0.51
CP(0.2)= 1.37 ± 0.5
CP(0.3)= 1.2 ± 0.26

Baek et al. 2023	*n/a*	*RCT*	*C57BL/ 6 mice*	*8w*	*n/a*	*3w*	*HFD*	Fish (tilapia) skin	*F/B ratio*<br>*PLA= 10.41 ± 4.01*<br>*CP= 5.26 ± 1.33*
**Wang et al. 2020**	8	PGD	C57BL/ 6J mice	10w	0.8 g/kg	8w	HFD	Walleye pollock skin	Body weight (g)<br>PLA= 36.09 ± 1.39<br>CP= 31.02 ± 1.05<br>Food intake (g/d)<br>PLA= 2.5 ± 0.3<br>CP= 2.24 ± 0.19<br>Adipocyte size (μm²)<br>PLA=4316.33 ± 377.55<br>CP=2193.88 ± 193.88<br>Subcutaneous fat (g)<br>PLA= 1.15 ± 0.12<br>CP= 0.72 ± 0.09<br>Epididymal fat (g)<br>PLA= 1.82 ± 0.14<br>CP= 0.49 ± 0.11<br>Liver (g)<br>PLA= 1.2 ± 0.06<br>CP= 1 ± 0.6<br>Cholesterol (mmol/L)<br>PLA= 10.09 ± 0.57<br>CP= 7.74 ± 0.71<br>HDL (mmol/L)<br>PLA= 1.5 ± 0.19<br>CP= 1.98 ± 0.26<br>LDL (mmol/L)<br>PLA= 1.15 ± 0.23<br>CP= 0.55 ± 0.17<br>Triacylglycerol (mmol/L)<br>PLA= 1.48 ± 0.19<br>CP= 0.73 ± 0.16<br>*F/B ratio*<br>*PLA= 0.57*<br>*CP= 1.5*
**Astre et al. 2018**	6	PGD	C57BL/ 6J mice	n/a	4 g/kg	20w	HFD	Fish collagen	Body weight (g)<br>PLA= 50.88 ± 3.01<br>CP= 45.88 ± 5.36<br>Energy intake (kcal/g of bw/d)<br>PLA= 1.6 ± 0.12<br>CP= 1.49 ± 0.15<br>Total body fat (g)<br>PLA= 20.19 ± 1.94<br>CP= 16.7 ± 3.48<br>Subcutaneous adipose tissue (g)<br>PLA= 3.07 ± 0.27<br>CP= 2.23 ± 0.51<br>Liver (g)<br>PLA= 2.18 ± 0.44<br>CP= 1.57 ± 0.47<br>Insulin (pg/mL)<br>PLA= 1903.88 ± 848.94<br>CP= 1377.08 ± 611.25<br>Glucose (mg/dL)<br>PLA= 132.58 ± 18.81<br>CP= 118.41 ± 10.12<br>Cholesterol (mmol/L)<br>PLA= 3.55 ± 0.64

									CP= 2.68 ± 0.32<br>Triacylglycerol (mmol/L)<br>PLA= 0.94 ± 0.15<br>CP= 1.15 ± 0.1
**Watanabe et al. 2021**	5	PGD	ddY mice	8w	n/a	3w	HFD	Chicken feet	Body weight (g)<br>PLA= 56.7 ± 8.27<br>CP= 56 ± 6.04<br>Visceral fat (g)<br>PLA= 4.25 ± 1.16<br>CP= 3.22 ± 0.89<br>Liver (g)<br>PLA= 1.88 ± 0.27<br>CP= 2.09 ± 0.42<br>Glucose (mg/100mL)<br>PLA= 93 ± 20.12<br>CP= 80 ± 6.71<br>Cholesterol (mg/100mL)<br>PLA= 170 ± 38.01<br>CP= 164 ± 35.78<br>Triacylglycerol (mg/100mL)<br>PLA= 59 ± 11.18<br>CP= 51 ± 15.65<br>(Gene expr.) Adiponectin (% of NC)<br>PLA= 1.2 ± 0.67<br>CP= 1.36 ± 1.1<br>(Gene expr.) Leptin (% of NC)<br>PLA= 3.04 ± 1.19<br>CP= 1.93 ± 1.03<br>(Gene expr.) IL-6 (% of NC)<br>PLA= 0.92 ± 0.47<br>CP= 0.57 ± 0.22<br>(Gene expr.) TNFα (% of NC)<br>PLA= 2.01 ± 0.8<br>CP= 1.54 ± 0.98<br>(Gene expr.) PPARγ (% of NC)<br>PLA= 0.93 ± 0.26<br>CP= 1.01 ± 0.72<br>(Gene expr.) SREBP1c (% of NC)<br>PLA= 0.63 ± 0.34<br>CP= 0.76 ± 0.47
**Koyama et al. 2013**	8	PGD	Crlj:CD (Sprague Dawley) rats	5w	0.2 g/kg	4w	HFD+HS	Nippi Inc., Tokyo, Japan. Source n/a	Body weight (g)<br>PLA= 433.79 ± 29.68<br>CP= 426.94 ± 26.26<br>Food intake (g/week)<br>PLA= 169.13 ± 14.81<br>CP= 171.98 ± 25.63<br>Cholesterol (mg/dL)<br>PLA= 87.89 ± 14.68<br>CP= 68.17 ± 9.58<br>HDL (mg/dL)<br>PLA= 27.51 ± 4.43<br>CP= 24.76 ± 2.75<br>LDL (mg/dL)<br>PLA= 9.32 ± 2.45<br>CP= 6.88 ± 2.14<br>Triacylglycerol (mg/dL)<br>PLA= 150.84 ± 53.87<br>CP= 140.07 ± 55.22

**Woo et al. 2020**	9	RCT	C57BL/ 6J mice	n/a	0.1/0.2/ 0.3 g/kg	8w	HFD	Skate (Raja kenojei) skin	Body weight (g)<br>PLA= 36.6 ± 2.7<br>CP(0.1)= 34.2 ± 2.1<br>CP(0.2)= 33.6 ± 2.9<br>CP(0.3)= 33.3 ± 2.6<br>Food intake (g)<br>PLA= 2.1 ± 0<br>CP(0.1)= 1.9 ± 0.1<br>CP(0.2)= 2 ± 0.1<br>CP(0.3)= 1.9 ± 0.1<br>Insulin (ng/mL)<br>PLA= 0.73 ± 0.15<br>CP(0.1)= 0.53 ± 0.08<br>CP(0.2)= 0.51 ± 0.07<br>CP(0.3)= 0.5 ± 0.02<br>Glucose (nmol/mL)<br>PLA= 0.73 ± 0.16<br>CP(0.1)= 0.53 ± 0.03<br>CP(0.2)= 0.52 ± 0.02<br>CP(0.3)= 0.5 ± 0.01<br>(Protein expr.) IL-6 (% of NC)<br>PLA= 1.14 ± 0.11<br>CP(0.1)= 1.03 ± 0.16<br>CP(0.2)= 0.99 ± 0.13<br>CP(0.3)= 0.82 ± 0.22<br>(Protein expr.) NFkB (% of NC)<br>PLA= 1.2 ± 0.28<br>CP(0.1)= 1.24 ± 0.32<br>CP(0.2)= 1.18 ± 0.39<br>CP(0.3)= 1.3 ± 0.39<br>plasma ROS (Flu/min/ul)<br>PLA=115.06 ± 39.38<br>CP(0.1)= 101.93 ± 38.61<br>CP(0.2)=78.38 ± 27.03<br>CP(0.3)=72.97 ±16.99<br>(Protein expr.) SOD (% of NC)<br>PLA= 0.53 ± 0.1<br>CP(0.1)= 0.86 ± 0.28<br>CP(0.2)= 0.93 ± 0.24<br>CP(0.3)= 0.96 ± 0.23<br>(Protein expr.) GPx (% of NC)<br>PLA= 0.55 ± 0.11<br>CP(0.1)= 0.73 ± 0.12<br>CP(0.2)= 0.88 ± 0.2<br>CP(0.3)= 0.9 ± 0.25<br>(Protein expr.) CAT (% of NC)<br>PLA= 0.79 ± 0.05<br>CP(0.1)= 0.82 ± 0.11<br>CP(0.2)= 0.85 ± 0.36<br>CP(0.3)= 0.86 ± 0.23<br>GSH (mM/g liver)<br>PLA=203.32 ±123.48<br>CP(0.1)=282.16 ± 58.09<br>CP(0.2)=282.16 ± 58.09<br>CP(0.3)=398.34 ± 53.94<br>TBARS (nM/mg liver)<br>PLA= 232.3 ± 44.1<br>CP(0.1)= 226.09 ± 39.13<br>CP(0.2)= 198.14 ± 35.4

									CP(0.3)= 180.12 ± 24.84
**Kim et al. 2009**	6	RCT	Sprague Dawley rats	5w	10% of whole diet	5w	HFD	Flathead Mullet (Mugil cephalus)	Δ Body weight (g)<br>PLA= 152.1±15.4<br>CP= 155.4±13.8<br>Food intake (g)<br>PLA= 464.8±17.2<br>CP= 471.1±18.8<br>Liver (g/ 100g bw)<br>PLA= 3.67±0.10<br>CP= 3.46±0.12<br>Cholesterol (mg/dL)<br>PLA= 133.8±4.2<br>CP= 99.7±2.8<br>HDL (mg/dL)<br>PLA= 20.4±0.8<br>CP= 22.5±1.8<br>LDL (mg/dL)<br>PLA= 81.7±2.0<br>CP= 72.7±1.7<br>Triacylglycerol (mg/dL)<br>PLA= 117.7±2.1<br>CP= 89.7±2.7<br>Glucose (mg/dL)<br>PLA= 167.3±3.1<br>CP= 150.7±3.9
**Duan et al. 2022**	10	RCT	Sprague Dawley rats	10w	3.4/ 2.05/ 0.68 g/kg	8w	n/a	Sheep bone	Body weight (g)<br>PLA= 373,75 ± 20<br>CP(0.68)= 339.58 ± 25.84<br>CP(2.05)=334.58 ± 27.5<br>CP(3.4)= 338.75 ± 26.25<br>Liver index (%)<br>PLA= 32.14 ± 2.36<br>CP(0.68)= 26.64 ± 3.23<br>CP(2.05)= 26.72 ± 2.1<br>CP(3.4)= 28.12 ± 6.46<br>MDA (nmol/mg protein)<br>PLA= 8.4 ± 0.47<br>CP(0.68)= 7.39 ± 0.36<br>CP(2.05)= 5.66 ± 0.35<br>CP(3.4)= 4.59 ± 0.22<br>SOD (U/mg protein)<br>PLA=363.2 ± 12.59<br>CP(0.68)=663.44 ± 10.65<br>CP(2.05)=560.77 ± 12.59<br>CP(3.4)=443.58 ± 14.53
**Lee et al. 2017**	8	RCT	ICR mice	6w	0.3 g/kg	8w	HFD	Tuna skin scale	Body weight (g)<br>PLA= 48.99 ± 5.06<br>CP= 41.05 ± 1.33<br>Epididymal adipocyte size (μm)<br>PLA= 0.37 ± 0.06<br>CP= 0.25 ± 0.03<br>Cholesterol (mg/dL) (n=6)<br>PLA= 224.5 ± 8.08<br>CP= 200.33 ± 9.87<br>HDL (mg/dL) (n=6)<br>PLA= 82.83 ± 11.44<br>CP= 98.33 ± 10.36

									LDL (mg/dL) (n=6)<br>PLA= 24.82 ± 3.89<br>CP= 21.23 ± 3.38<br>Triacylglycerol (mg/dL) (n=6)<br>PLA= 200.17 ± 12.3<br>CP= 188.5 ± 10.51<br>(Protein expr.) PPARγ (% of NC)<br>PLA= 28.47 ± 9.3<br>CP= 4,14 ± 2.7<br>(Protein expr.) C/EBPα (% of NC)<br>PLA= 2.9 ± 0.28<br>CP= 1.09 ± 0.36
**Zhang et al. 2022**	8	RCT	C57BL/6J mice	7w	0.05 g/kg	8w	HFD	Jellyfish (Nemopilema nomurai)	ΔBody weight (g)<br>PLA=6.73 ± 3.11<br>CP=1.26 ± 1.75<br>Glucose (mM)<br>PLA= 8.41 ± 2.72<br>CP= 6.4 ± 1.92<br>Cholesterol (mM)<br>PLA= 3.98 ± 1.81<br>CP= 2.26 ± 1.84<br>Triacylglycerol (mM)<br>PLA= 1.94 ± 0.91<br>CP= 0.9 ± 0.42<br>IL-1β (pg/mL)<br>PLA= 36.23 ± 19.71<br>CP= 25 ± 11.12<br>TNFα (pg/mL)<br>PLA= 141.67 ± 84.46<br>CP= 82.64 ± 38.3<br>ROS (% of NC) (n=3)<br>PLA= 272.87 ± 74.51<br>CP= 177.85 ± 64.5<br>GPx (nmol/min per mg protein)<br>PLA= 27.27 ± 18.19<br>CP= 46.87 ± 31.93<br>GSH (nmol/mL)<br>PLA= 194.35 ± 67.8<br>CP= 273.97 ± 142.86
**Tometsuka et al. 2021**	10	PGD	BALB/c mice	6w	4 %N of control diet	10w	NC	Bovine bone	ΔBody weight (g)<br>PLA=5.1 ± 1.5<br>CP=5.6 ± 1.4<br>Food intake (g)<br>PLA= 248.2 ± 28.6<br>CP= 267.5 ± 18.3<br>Adipocyte size (µm²)<br>PLA= 1201.2 ± 144.7<br>CP= 1424.1 ± 272<br>Cholesterol (mg/L)<br>PLA= 1250.85 ± 81.36<br>CP= 1122.03 ± 50.85<br>Triacylglycerol (mg/L)<br>PLA= 733.63 ± 401.79<br>CP= 437.67 ± 127.35<br>(Gene expr.) FAS (% of PLA)<br>PLA= 1 ± 0.88<br>CP= 0.41 ± 0.11

**Kalmukova et al. 2023**	20	RCT	Wistar rats	9w	1 g/kg	6w	HCD	Scales of the mackerel icefish (Champsocephalus gunnari)	ΔBody mass (%)<br>PLA= 271 ± 76.03<br>CP=238 ± 102.86<br>Food intake (kcal/g/d)<br>PLA= 0.59 ± 0.27<br>CP= 0.48 ± 0.22<br>Visceral fat (%)<br>PLA= 2.9 ± 1.34<br>CP= 1.78 ± 0.13<br>Subcutaneous fat (%)<br>PLA= 1.9 ± 0.89<br>CP= 1.34 ± 0.36<br>Visceral adipocyte size (μm²)<br>PLA= 7294.49 ± 1070.5<br>CP= 5593.7 ± 901.45
Guo et al. 2022	*8*	*RCT*	*C57BL/ 6J mice*	*7w*	*0.5/1/2 g/kg*	*12w*	*HFD*	Yak (Bos grunniens)	*F/B ratio*<br>*PLA= 6.75*<br>*CP(2)= 1.64*
**Watanabe et al. 2022**	8	PGD	C57BL/ 6J mice	6w	2.5/5 %	4w	HFD	Unfermented (uCP=5%; taken for meta-analysis) & Fermented (fCP2.5/5%) porcine collagen	Body weight (g)<br>PLA= 27.2 ± 1.13<br>fCP(5)= 24.2 ± 0.85<br>fCP(2.5)= 25.3 ± 1.13<br>uCP= 26.3 ± 1.41<br>Visceral fat (g)<br>PLA= 1.53 ± 0.14<br>fCP(5)= 1.11 ± 0.17<br>fCP(2.5)= 1.37 ± 0.14<br>uCP= 1.37 ± 0.25<br>Liver (g)<br>PLA= 1.13 ± 0.06<br>fCP(5)= 0.87 ± 0.08<br>fCP(2.5)= 0.94 ± 0.08<br>uCP= 0.97 ± 0.08<br>Adiponectin (ng/mL)<br>PLA= 419 ± 48.08<br>fCP(5)= 1290 ± 104.65<br>fCP(2.5)= 662 ± 132.94<br>uCP= 488 ± 84.85<br>Leptin (pg/mL)<br>PLA= 8713 ± 1083.29<br>fCP(5)= 2961 ± 523.26<br>fCP(2.5)= 5541 ± 701.45<br>uCP= 5848 ± 523.26
**Raksha et al. 2018**	10	RCT	Wistar rats	13w	1 g/kg	6w	HCD	Wild marine fish scales	Body weight (g)<br>PLA= 463.41 ± 8.41<br>CP= 401.49 ± 7.4<br>Food intake (g)<br>PLA= 31 ± 1.5<br>CP= 25 ± 1.3<br>HbA1c (μmol fructose/g hemoglobin)<br>PLA= 0.81 ± 0.06<br>CP= 0.52 ± 0.06<br>Insulin (%)<br>PLA= 159.8 ± 7.5<br>CP= 105.3 ± 6.5<br>Glucose (mmol/L)<br>PLA= 7.3 ± 0.5<br>CP= 5.2 ± 0.5<br>IL-1β (% of NC)

									PLA= 137.01 ± 8.77<br>CP= 116.88 ± 6.82<br>IL-4 (% of NC)<br>PLA= 107.02 ± 4.16<br>CP= 107.02 ± 6.12<br>IL-10 (% of NC)<br>PLA= 106.48 ± 4.23<br>CP= 118.9 ± 5.81<br>IL-12 (% of NC)<br>PLA= 137.7 ± 6.15<br>CP= 106.93 ± 2.93<br>MDA (nmol/mg protein)<br>PLA= 501.95 ± 25.39<br>CP= 388.67 ± 15.63<br>SOD (U/min/mg protein)<br>PLA= 23.7 ± 3.91<br>CP= 33.07 ± 4.69<br>CAT (mmol H_2O_2/min/mg protein)<br>PLA= 45.5 ± 11.37<br>CP= 123.7 ± 25.59
**Wang et al. 2008**	10	PGD	Sprague Dawley rats	n/a	1/3/9 g/kg	6 w	HFD	Marine collagen peptides	Cholesterol (mmol/L)<br>PLA= 3.37 ± 0.24<br>CP(1)= 1.89 ± 0.29<br>CP(3)= 2.07 ± 0.39<br>CP(9)= 1.99 ± 0.29<br>LDL (mmol/L)<br>PLA= 2.2 ± 0.34<br>CP(1)= 0.83 ± 0.16<br>CP(3)= 1.01 ± 0.35<br>CP(9)= 0.91 ± 0.26<br>Triacylglycerol (mmol/L)<br>PLA= 2.2 ± 0.34<br>CP(1)= no data<br>CP(3)= 0.9 ± 0.15<br>CP(9)= 0.86 ± 0.12<br>MDA (nmol/mL)<br>PLA= 15.9 ± 9.9<br>CP(1)= no data<br>CP(3)= no data<br>CP(9)= 7.1 ± 4.1<br>SOD (U/mL)<br>PLA= 119.7 ± 47.8<br>CP(1)= 218.6 ± 33.2<br>CP(3)= 242.7 ± 21.4<br>CP(9)= 242.1 ± 47.8
**Miao et al. 2022**	8	RCT	C57BL/6J mice	9w	0.1/0.2 g/kg	6w	HFD	Skin of Monkfish (Lophius litulon)	Body weight (g)<br>PLA= 30.8 ± 0.59<br>CP(0.1)= 26.5 ± 0.67<br>CP(0.2)= 24.85 ± 0.94<br>Liver index (%)<br>PLA= 6.38 ± 0.09<br>CP(0.1)= 5.58 ± 0.02<br>CP(0.2)= 4.92 ± 0.02<br>Cholesterol (mmol/L)<br>PLA= 7.24 ± 1.43<br>CP(0.1)= 4.44 ± 0.38<br>CP(0.2)= 3.66 ± 0.31<br>Triacylglycerol (mmol/L)<br>PLA= 4.01 ± 0.1

CP(0.1)= 2.09 ± 0.45<br>CP(0.2)= 1.66 ± 0.38<br>IL-1β (pg/mL)<br>PLA= 91.65 ± 3.05<br>CP(0.1)= 49.59 ± 3.23<br>CP(0.2)= 33.20 ± 2.27<br>IL-6 (pg/mL)<br>PLA= 68.45 ± 2.75<br>CP(0.1)= 41.16 ± 2.22<br>CP(0.2)= 28.91 ± 1.73<br>TNFα (pg/mL)<br>PLA= 110.09 ± 3.79<br>CP(0.1)= 76.96 ± 3.34<br>CP(0.2)= 56.19 ± 3.84<br>MDA (nmol/mg protein)<br>PLA= 16.59 ± 1.11<br>CP(0.1)= 12.38 ± 0.82<br>CP(0.2)= 10.59 ± 0.78<br>SOD (U/mg protein)<br>PLA= 67.85 ± 10.43<br>CP(0.1)= 96.91 ± 9.49<br>CP(0.2)= 132.78 ± 8.86<br>GPx (U/mg protein)<br>PLA= 139.18 ± 10.43<br>CP(0.1)= 160.38 ± 11.54<br>CP(0.2)= 210.87 ± 5.28<br>CAT (U/mg protein)<br>PLA= 7.53 ± 1.25<br>CP(0.1)= 11.63 ± 1.35<br>CP(0.2)= 13.09 ± 0.66

n/a = not available, RCT = randomized controlled trial, PGD = parallel group design (randomization unclear), SOD = superoxide dismutase activity, TBARS = Thiobarbituric acid reactive substances, w = weeks, HCD = high-caloric diet, HFD = high-fat diet, HS = high sucrose, WAT = white adipose tissue, GAPDH = glyceraldehyde-3-phosphate dehydrogenase, C/EBPα = CCAAT/enhancer-binding protein alpha, NC = normal control (no HF/HC diet), bw = body weight, n/a = not available, HDL = high-density lipoprotein, LDL = Low-density lipoprotein, PPARα/γ = peroxisome proliferator-activated receptor alpha/gamma, SREBP = sterol regulatory element binding protein, TNFα = tumour necrosis factor alpha, HS = high sucrose, IL- = interleukine, ROS = reactive oxygen species, GPx = glutathione peroxidase, CAT = catalase, GSH = glutathione, MDA = malondialdehyde, liver index = usually calculated as liver weight/bw x 100%, italics = not included in meta-analysis but systematic review due to missing SD's or mean values (F/B ratios).
